# Study of the Electrochemical Behavior of N-Substituted-4-Piperidones Curcumin Analogs: A Combined Experimental and Theoretical Approach

**DOI:** 10.3390/ijms232315043

**Published:** 2022-11-30

**Authors:** John Amalraj, Claudia E. Vergara, Matías Monroy-Cárdenas, Ramiro Araya-Maturana, Maximiliano Martínez-Cifuentes

**Affiliations:** 1 Instituto de Química de Recursos Naturales, Universidad de Talca, Talca 3460000, Chile; 2 Departamento de Ciencias Básicas, Facultad de Ciencias, Universidad Santo Tomás, Avda. Carlos Schorr 255, Talca 8370003, Chile; 3 MIBI: Interdisciplinary Group on Mitochondrial Targeting and Bioenergetics, Universidad de Talca, P.O. Box 747, Talca 3460000, Chile; 4 Departamento de Química Orgánica, Facultad de Ciencias Químicas, Universidad de Concepción, Edmundo Larenas 129, Concepción 4070371, Chile

**Keywords:** curcumin, electrochemistry, DFT, curcuminoids, piperidones, benzyl derivatives

## Abstract

The electrochemical behavior of N-methyl- and N-benzyl-4-piperidone curcumin analogs were studied experimentally and theoretically. The studied compounds present different substituents at the *para* position in the phenyl rings (-H, -Br, -Cl, -CF_3_, and -OCH_3_). We assessed their electrochemical behavior by differential pulse and cyclic voltammetry, while we employed density functional theory (DFT) M06 and M06-2x functionals along with 6-311+G(d,p) basis set calculations to study them theoretically. The results showed that compounds suffer a two-electron irreversible oxidation in the range of 0.72 to 0.86 V, with surface concentrations ranging from 1.72 × 10^−7^ to 5.01 × 10^−7^ mol/cm^2^. The results also suggested that the process is diffusion-controlled for all compounds. M06 DFT calculations showed a better performance than M06-2x to obtain oxidation potentials. We found a good correlation between the experimental and theoretical oxidation potential for N-benzyl-4-piperidones (R^2^ = 0.9846), while the correlation was poor for N-methyl-4-piperidones (R^2^ = 0.3786), suggesting that the latter suffer a more complex oxidation process. Calculations of the BDEs for labile C-H bonds in the compounds suggested that neither of the two series of compounds has a different tendency for a proton-coupled electron transfer (PCET) oxidation process. It is proposed that irreversible behavior is due to possible dimerization of the compounds by Shono-type oxidation.

## 1. Introduction

Curcumin (diferuloylmethane), a yellow compound isolated from the turmeric plant (Curcuma longa), is one of the most extensively studied naturally occurring polyphenols. The pharmacological properties of curcumin, related to a large variety of diseases, have attracted great interest [1,2,3,4,5,6,7]. Despite these interesting pharmacological properties, it suffers from several drawbacks for practical applications, due to its poor bioavailability, which is a consequence of low chemical stability and low water solubility [8,9,10]. The latter has prompted the search for curcumin analogs that keep or improve their bioactivity, while, at the same time, overcoming their drawbacks. Several structural modifications have been afforded to enhance the bioavailability of curcumin [11,12,13]. It has been proposed that compounds possessing a β-diketone moiety, as in the case of curcumin, are a substrate of liver aldo-keto reductases, which probably contribute to their rapid in vivo metabolism [12,14,15]. To avoid this drawback, multiple synthetic alternatives have been assayed, among which replacing the β-diketone moiety with a heterocyclic ring has shown to be effective [12,16]. Among the alternatives, synthetic analogs of curcumin possessing a 4-piperidone scaffold have shown high bioactivity, e.g., as anti-inflammatory and anticancer agents [17,18,19,20,21].

One of the aspects of curcumin that have presented some controversy is its electrochemical behavior [22,23,24,25]. During recent years, the electrochemical behavior of some of the derived curcuminoid families also have been studied, e.g., the electrochemistry of pyrazole derivatives of curcumin [26], the electrochemistry and antioxidant capacity of curcumin derivatives obtained by esterification of its phenol groups [27], and the electronic and conductance properties of a couple of thiophene curcuminoids using electrochemical techniques, ultraviolet spectroscopy, and DFT calculations [27]. However, until now, the electrochemical behavior of N-substituted-4-piperidone curcumin analogs has not been reported, which seems relevant to study due to their interesting pharmacological properties, which have been suggested to be associated with the electron transfer process [13]. Besides our previous work on the reactivity of these compounds, only theoretical work dealing with the potential technological application for the non-linear optics of this type of curcuminoid can be found [16,28].

Based on the above, in this work we propose to study the electrochemical behavior of N-substituted-4-piperidone curcuminoid analogs (Table 1). The effect of different substituents on the aromatic rings and the variation in the N-substituent of the piperidone ring (methyl and benzyl) could help in the understanding of the electrochemical behavior and help in the design of curcumin piperidone derivatives with biological activity, besides determining the oxidation potentials that could be useful in obtaining derivatives by electrochemical synthesis [29,30,31]. We employ differential pulse and cyclic voltammetry to experimentally study the electrochemistry of compounds, and DFT calculations to obtain theoretical redox potentials, which are then compared with those obtained experimentally, assessing the effects of the substituents at the phenyl substituent.

## 2. Results and Discussion

### 2.1. Synthesis

Compounds were obtained by the base-catalyzed aldolic condensation of N-methyl or N-benzyl-4-piperidones with the corresponding aldehyde (Table 2). Moderate to high yields were achieved, ranging from 48% for compound **C9** to 88% for compound **C5**. Compounds **C1** to **C8** and **C10** have been previously described (see references indicated in Table 2), while compound **C9** is described here for the first time. 

### 2.2. Differential Pulse Voltammetry (DPV) Results

**Figure 1:**
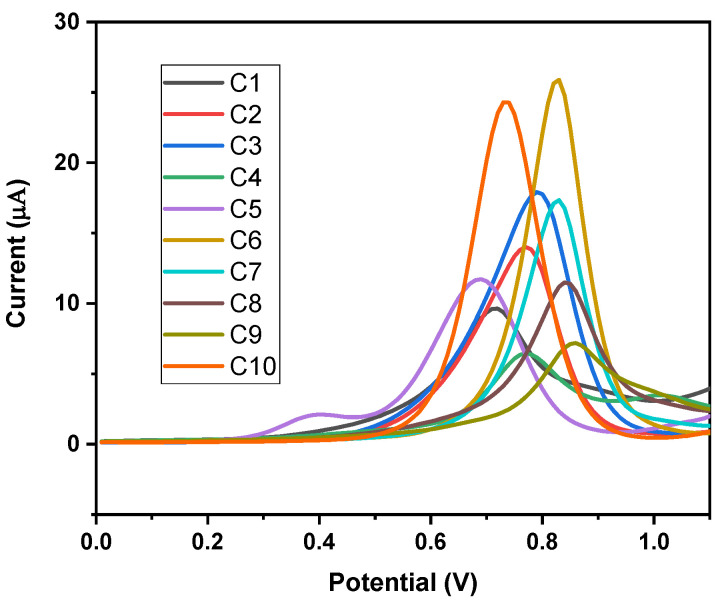
DP voltammograms of 2.0 mM solutions of compounds. Non-aqueous medium: 0.1 M of TBAP in CH_3_CN.

DPV results reveal that derivatives exhibited one well-defined anodic peak at potentials higher than +0.7 V versus Ag/AgCl (non-aqueous) (Figure 1 and Table 3). Oxidation peak potential values for the derivatives with electron-withdrawing groups were shifted towards more positive values, whereas derivatives with electron-releasing groups were shifted towards negative values. Similarly, when we compare the oxidation peak potential of N-methyl-substituted derivatives (**C1** to **C5**) with N-benzyl-substituted derivatives (**C6** to **C10**), the latter shifted the oxidation potential towards more positive values.

### 2.3. Number of Electrons (n) Calculation

Our results showed that by increasing the scan rate, the peak potential is shifted to more positive potentials. Laviron’s equation was used to estimate n as follows [32]:

Ep = E^0^ + (RT/αnF)[ln(RTk_s_/αnF) − lnυ], (1)

where E is the potential; E⁰ is the formal standard potential; ks is the standard heterogeneous reaction rate constant; n is the transfer electron number; α refers to the charge-transfer coefficient; υ is scan rate; R is the gas constant; T is the temperature; and F is the Faraday constant. When plotting the graph between Ep versus lnυ, the slope gives the value of RT/αnF (α = 0.5); from this, we can calculate the number of electrons involved in the process (Table 4). In this way, n was found to be 2, implying that two electrons were transferred in the electrochemical redox reaction.

### 2.4. Surface Concentration 

The surface concentrations of piperidone derivatives (Γ) were calculated employing the following equation:

I = n^2^F^2^AΓυ/4RT, (2)

where I is the peak current; n = 2 (number of electrons involved in the electrochemical process); F is the Faraday constant 96,485 C mol^−1^; A is the surface area, 0.0707cm^2^ (the diameter of the working electrode, the glassy carbon electrode, is 0.3 cm, and from this, the surface area of the electrode was calculated); υ represents the scan rate; R is the gas constant, 8.314 JK^−1^mol^−1^; and T = 293 K. If we plot the graph of Ip versus the scan rate, which conforms to the following equation: Ip (μA) = 0.721υ + 10.223 (R^2^ = 0.9153), in this equation the slope, gives the value of n^2^F^2^AΓ/4RT, from which we can calculate Γ. The results are presented in Table 4.

In the electrochemical process, there is an important difference between the concentration of a species at the surface of the electrode and its concentration at some distance from it, generally known as bulk concentration. The surface concentration depends on the rate at which the reactants are brought to the electrode surface by either diffusion or flow processes, which determines the rate of the electrochemical reaction.

All compounds exhibit a surface concentration in the order of 10^−7^ mol/cm^2^, compound **C4** (N-methyl-4-piperidone with trifluoromethyl in phenyl rings) has the lowest value (1.72 × 10^−7^), and compound **C6** (N-benzyl-4-piperidone unsubstituted in phenyl rings) has the highest value (5.01 × 10^−7^).

### 2.5. Cyclic Voltammetry Characterization 

**Figure 2:**
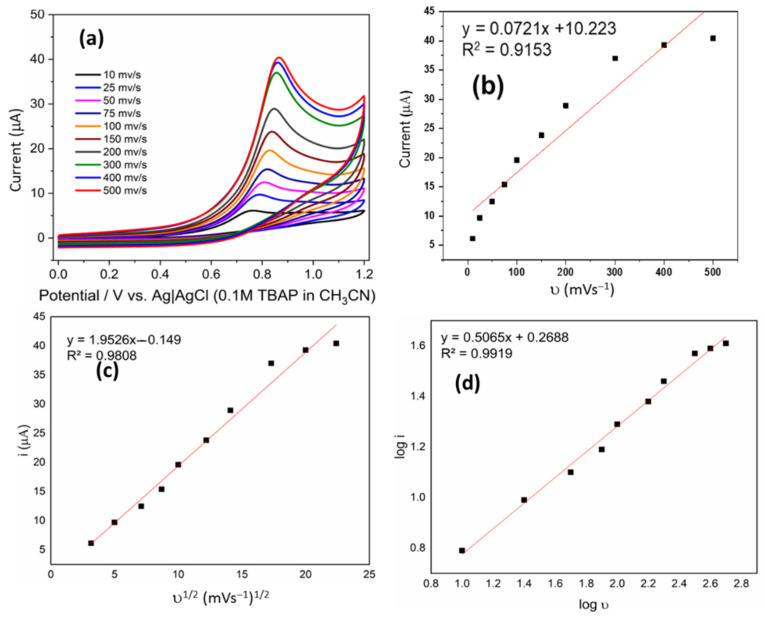
(**a**) Cyclic voltammograms of 2 mM of **C1** in 0.1 M solution of TBAP in CH_3_CN at different scan rates. (**b**) Linear relationship between i and υ. (**c**) Linear relationship between i and υ^1/2^ (**d**) Linear relationship between log i and log υ.

**Figure 3:**
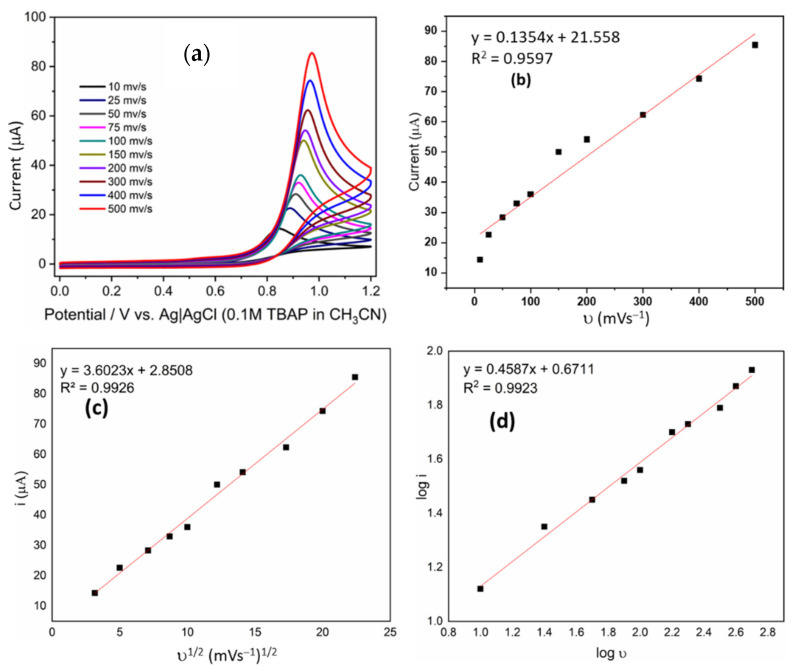
(**a**) Cyclic voltammograms of 2 mM of **C6** in 0.1 M solution of TBAP in CH_3_CN at different scan rates. (**b**) Linear relationship between i and υ. (**c**) Linear relationship between i and υ^1/2^ (**d**) Linear relationship between log i and log υ.

Scan rate has a great influence on the redox process of the electrode surface. Therefore, cyclic voltammograms of 2 mM of the derivatives were recorded in the scan rate range from 10 to 500 mVS^−1^ and the peak current increased with increased scan rate for all compounds (Figure 2a and Figure 3a). The linear relationships of the square root of the scan rate and the scan rate on the peak current are shown in Figure 2c and Figure 3c.

The dependence between I_p_ and ν shows a good linear correlation; the relationship can be presented by the following equation: Ip (μA) = 0721ν (V/s) + 10.223 (R^2^ = 0.9153) (Figure 2b). However, I_p_ on ν^1/2^ has better linear correlation, whose relationship can be presented by the following equation: Ip (μA) = 1.9526 (V^1/2^/s^1/2^) − 0.149 (R^2^ = 0.9808) (Figure 2c). A similar analysis was performed for the compound 7, which shows Ip (μA) = 0.1354ν (V/s) + 21.558 (R^2^ = 0.9597) (Figure 3b), and for i_p_ on ν^1/2^, Ip (μA) = 3.6023 (V^1/2^/s^1/2^) + 2.8508 (R^2^ = 0.9926) (Figure 3c). These observations strongly suggest that redox reactions of both derivatives are diffusion-controlled. Similar analyses were performed for all derivatives, and similar behavior was found in all cases. 

Figure 2d and Figure 3d shows the relationship between the logarithm of redox peak current and the logarithm of scan rate, which conformed to the following equation: log Ip (A) = 0.5065 Log ν (V/s) + 0.2688 (R^2^ = 0.9919). As is known, the slope close to 0.5 is ascribed to a diffusion-controlled process, whereas the slope close to 1.0 is ascribed to an adsorption-controlled process. 

### 2.6. Quantum Chemical Calculation

The oxidation potentials at DFT M06 and M06-2x level using 6-311+G(d,p) basis set were obtained for the ten derivatives. M06-2X functionals have been extensively used to calculate redox potentials [33,34,35,36,37]; however, in the specific case of oxidation potential, previous work has shown that functionals with high levels of Hartree–Fock (HF) exchange percentage tend to significantly overestimate the potential [38]. Therefore, we tested M06-2X (54% HF) and M06 functionals to obtain the potentials. The number of electrons transferred in the reactions, according to Laviron’s equation in Section 3.2, was two. Accordingly, we considered the two possible electronic states for oxidized species (singlet and triplet dications) for the calculations. Table 5 and Table 6 show the values for experimental and theoretical oxidation potentials calculated with M06-2X and M06 functionals, respectively. In all cases, the mean absolute error (MAE) for M06 was lower than for M06-2X, which agrees with what has been previously reported [38]. MAE values are still overestimated with the M06 functional, but they are in the range of previous work of calculated oxidation potentials [39]. Based on the above, we use data obtained with the M06 functional to discuss the results.

Taking both series (N-methyl and N-benzyl) together, experimental and theoretical potentials did not correlate well, neither when considering the oxidized species as a triplet, nor as a singlet (R^2^ = 0.0796 for singlet, R^2^ = 0.1667 for triplet). However, when both series were treated separately, it was found that the series with the N-benzyl group at the piperidone ring presented a good correlation between experimental and theoretical oxidation potential.

**Figure 4:**
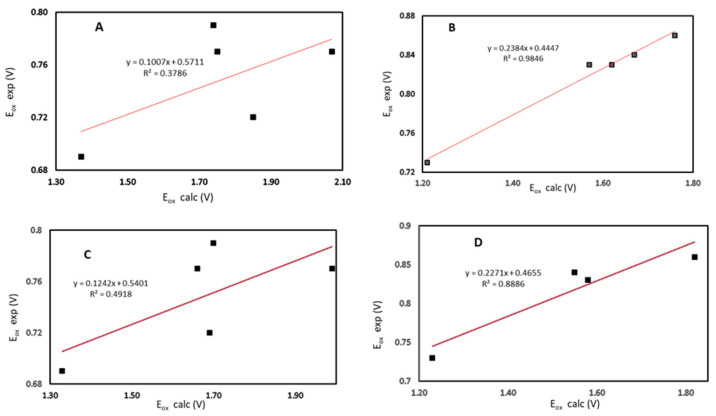
Experimental versus calculated oxidation potential for (**A**) compound **C1** to **C5** considering singlet oxidized species; (**B**) compound **C6** to **C10** considering singlet oxidized species; (**C**) compound **C1** to **C5** considering triplet oxidized species; (**D**) compound **C6** to **C10** considering triplet oxidized species.

Figure 4 presents the correlation between experimental and theoretical oxidation potential for the N-methyl (A) and N-benzyl (B) series considering the oxidized species as a singlet, and for the N-methyl (C) and N-benzyl (D) series considering the oxidized species as a triplet.

For the series with N-benzyl (compounds **C6** to **C10**), good correlations were found considering both kinds of oxidized species, triplet or singlet (R^2^ = 0.8886 and R^2^ = 0.9846, respectively). Meanwhile, the series with the N-methyl group did not show a good correlation between experimental and theoretical oxidation potentials, neither when considering the oxidized species as triplet nor as singlet (R^2^ = 0.4918 and R^2^ = 0.3786, respectively). 

Considering that the N-benzyl derivative series presented a good correlation between experimental and theoretical oxidation potentials, unlike that evidenced for the N-methyl series, it can be suggested that oxidation of this last series of compounds did not occur by a simple mechanism. The above indicates that variation of the substituent at nitrogen is a key factor that determines the redox behavior of these derivatives. In addition, based on the structural analysis mentioned above, it is reasonable to consider that the trend for both series will remain independent of the addition of more compounds for each series.

**Figure 5:**
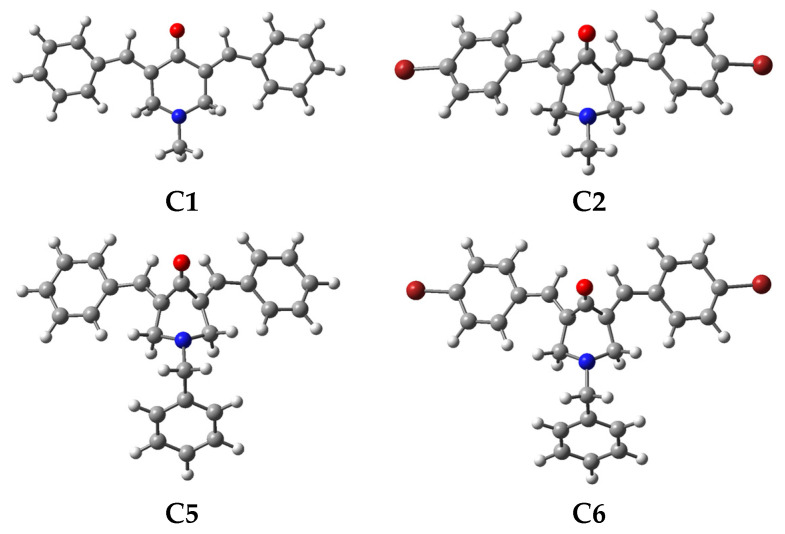
Optimized geometry at the M06/6-311+G(d,p) level for the oxidized species (singlet dication) of compound **C1**, **C2**, **C5**, and **C6**.

Triplet oxidized species for all compounds present a similar geometry, regardless of the substituent on the N-position or phenyl rings. However, for singlet oxidized species, the behavior varies between the N-methyl and N-benzyl series (Figure 5). For the N-methyl series, the oxidized species of compounds **C2**, **C3**, and **C5** (with p-Br, p-Cl, and p-OCH3 in the phenyl rings) present a notable geometry distortion of the piperidone ring, which does not occur for **C1** and **C4** (unsubstituted and with p-CF3 in the phenyl rings). On the other hand, all oxidized species of the N-benzyl series present a notable geometrical distortion of the piperidone ring, regardless of the substituent on the phenyl rings.

The geometry of the singlet and triplet oxidized species for all compounds are in Tables S1 and S2 of the Supplementary Materials. 

To obtain insights into the behavior observed for these two series of compounds, we studied the energy of frontier molecular orbitals. In previous works, theoretical calculations of some of these compounds had been carried out to study their reactivity and the behavior of their radical anions [16], as well as optical properties [28]. In our previous work [16], we obtained, at the B3LYP/6-31G(d) level, the energies of the frontier molecular orbitals of some compounds studied here (**C1**, **C2**, **C3**, **C6**, **C7**, and **C8**). Table 7 shows the values for HOMO and LUMO energies (E_HOMO_ and E_LUMO_, respectively) and the HOMO–LUMO energy gap (GAP_H-L_). These values, calculated at M06/6-311+G(d,p), showed slight differences from those calculated previously for some of the compounds at the B3LYP/6-31G(d) level. The E_HOMO_ values calculated at M06/6-311+G(d,p) tend to be lower, while the E_LUMO_ values tend to be higher, which leads to the GAP_H-L_ being lower for M06/6-311+G(d,p). However, the tendency for the three compounds in common for each series is the same. Unsubstituted derivatives in the aromatic ring show the highest E_HOMO_ and E_LUMO_, followed by *p*-Br and p-Cl (both for N-methyl and N-benzyl series). In addition, it can be observed that the value of GAP_H-L_ did not show significant differences among compounds from the N-methyl and N-benzyl series. 

**Figure 6:**
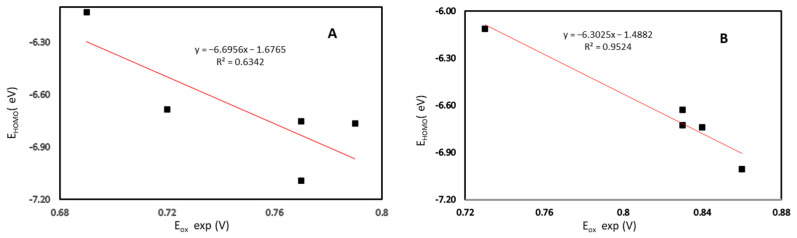
Experimental oxidation potential (E_ox_ exp) versus HOMO energy (E_HOMO_) for (**A**) compounds **C1** to **C5**, and (**B**) compounds **C6** to **C10**.

We examined the correlation among E_HOMO_ and experimental E_ox_ pairs, considering that electrons on this orbital are those that are removed in the oxidation process. Figure 6 shows the correlation for both the N-methyl and N-benzyl series. We found that the E_HOMO_ of N-benzyl derivatives correlates well with experimental E_ox_, with an R^2^ = 0.9524. On the other hand, N-methyl derivatives did not present a good correlation, obtaining an R^2^ = 0.6342. These results also support the assumption that the oxidation of the N-methyl derivatives, unlike the N-benzyl ones, suffers a complex oxidation process that does not only involve the direct subtraction of two electrons from the HOMO.

To evaluate alternative possible mechanisms, which can imply the heterolytic cleavage of a carbon–hydrogen (C-H) bond, we study the bond dissociation enthalpies (BDEs) of the C–H bonds potentially breakable in compounds **1** and **6** (BDE1 and BDE2 in Table 8). A significant difference between the lower BDE would be indicative of a possible differential mechanism between the compounds of both series. 

Results show that for both compounds, the BDE2 was the lowest, and there are no significant differences in the values (61.50 kcal/mol for **1** vs. 60.15 kcal/mol for **6**). The latter suggests that neither of the two series of compounds (N-methyl and N-benzyl derivatives) has a significant preference for the proton-coupled electron transfer (PCET) mechanism; therefore, this cannot explain the differences between both series.

A plausible explanation for the irreversible oxidation of the compounds is to consider a chemical reaction coupled to the electrochemical process. For heterocyclic tertiary amines, an electrochemical oxidation reaction (Shono oxidation) has been described that gives products with a substituent in the carbon vicinal to the nitrogen [30,40,41]. The process consists of a two-electron oxidation coupled to a proton transfer to generate an iminium cation intermediate which reacts with nucleophiles to achieve a great variety of products depending on the conditions [42,43,44]. In our case, it is possible that the attacking nucleophile corresponds to the same molecule generating a dimer. This hypothesis needs to be evaluated in future works that delve into mechanistic aspects of the oxidation of these compounds.

## 3. Materials and Methods

### 3.1. Synthesis

The synthesis of 4-piperidinone derivatives was performed in agreement with reported methods with slight modification (Table 1). To a solution of 1-methyl- or 1-benzyl-4-piperidone (0.30 g, 1.0 equivalent) in ethanol:water 1:1 (2 mL), KOH (0.3 g) was added and sonicated for 5 min. Then, the appropriate aldehyde (2.0 equivalents) was added, and the mixture was sonicated for 15 min at room temperature. The separated solid was filtered and washed with ethanol–water (20 mL) to obtain a yellow solid. Finally, product **C1** was crystallized from ethanol, compound **C5** was crystallized from methanol, and the other compounds were crystallized from a mixture of chloroform:methanol. Synthesis of the new compound (3E,5E)-1-benzyl-3,5-bis(4-(trifluoromethyl)benzylidene)piperidin-4-one (**C9**) was carried out as follows: to a solution of 1-benzyl) 4-piperidone (0.30 g, 1.59 mmol) in ethanol:water 1:1 (2 mL), KOH (0.3 g) was added and sonicated for 5 min. Then, 4-trifluoromethylbenzaldehyde (0.55 g, 3.18 mmol) was added and the mixture was sonicated for 15 min at room temperature. The separated solid was filtered and washed with ethanol–water (20 mL) to obtain a yellow solid (0.83 g, 52% yield). Finally, the product was purified by column chromatography using hexane:ethyl acetate 4:1. ^1^H RMN (400.13 MHz, CDCl_3_) δ: 3.71 (s, 2H), 3.84 (s, 4H), 7.24 (s, 5H), 7.42 (d, *J* = 8.1 Hz, 4H), 7.63 (d, *J* = 8.1, 4H), 7.80 (s, 2H). ^13^C RMN (100.61 MHz, CDCl_3_) δ: 54.1, 61.3, 123.9 (q, *J* = 271.8 Hz), 125.5 (q, *J* = 4.1), 127.6, 128.42, 128.9, 129.6, 130.2, 130.6 (q, *J* = 32.7), 134.9, 135.1, 136.9, 138.5, 187.27. M.p. 129.3–131.5 °C.

### 3.2. Electrochemical Experiments

Electrolytic medium: acetonitrile containing 0.1 M tetrabutylammonium perchlorate (TBAP) was used as the electrolytic medium and the working concentrations of each compound were 2 mM. 

Differential pulse voltammetry (DPV), cyclic voltammetry (CV), square wave voltammetry, and electrochemical impedance spectroscopy (EIS) were carried out with a CH Instrument (CHI 750) electrochemical workstation. All electrochemical experiments were carried out with 2.0 mM of each piperidone derivative. A stationary glassy carbon electrode (GCE, CH Instrument with an area of 0.0707 cm^2^) was used as the working electrode. The surface of the electrode was polished to a mirror finish with 0.1 μm alumina powder before each experiment, and the surface was cleaned with ethanol under an ultrasonication for 30 s. A platinum spiral wire was used as an auxiliary electrode and the potentials were measured against a non-aqueous Ag/Ag^+^ reference electrode CH Instrument 112. All experiments were performed in ambient conditions.

### 3.3. Computational Details

All calculations were carried out using Gaussian 09 [45] program package, revision a.01; Gaussian, Inc.: Wallingford, CT, USA). Geometries were calculated without symmetry constraints at the DFT M06 and M06-2X level with the 6-311+G (d,p) basis set. The conductor-like polarizable continuum (C-PCM), which has shown good performance to redox potential calculations [8], was used to include the solvent effect (acetonitrile). No imaginary vibrational frequencies were found at the optimized geometries, indicating that they are the true minima of the potential energy surface. 

In previous work [46], we applied a thermodynamic cycle to obtain the Gibbs free energy of the reaction in solution (G°(t)) for calculation of the redox potentials of quinones, and we used the same approach here. Reduction potentials concerning the Ag/AgCl reference electrode were obtained by the following equation:

(3) $$E_{red}=\frac{-\Delta G{}^{\circ}\left(t\right)}{nF}-4.72\;V$$

where the value 4.72 V corresponds to the redox potential of the Ag/AgCl couple. Bond dissociation enthalpies were calculated following a previous methodology [8,47].

## 4. Conclusions

In this work, the electrochemical behavior of N-substituted-4-piperidone curcumin analogs was studied experimentally and theoretically. Oxidation potentials ranged from 0.72 to 0.89 V, with N-methyl-4-piperidones values approximately 0.1 V lower than those of N-benzyl-4-piperidones. We found that the electrochemical oxidation of these compounds implies a two-electron transfer process. Cyclic voltammetry studies showed that compounds suffer irreversible oxidation and suggested that it is a diffusion-controlled process. DFT quantum chemical calculations with M06 and M06-2x functionals showed that the formed works better to calculate the oxidation potentials of these compounds. The results also suggested that the electronic state for oxidized species is a singlet. A good correlation between experimental and calculated oxidation potentials for N-benzyl-4-piperidones (r = 0.9846) showed that calculations were representative of the oxidation process. On the other hand, the calculated oxidation potentials for N-methyl-4-piperidones exhibited a poor correlation with the experimental values. The latter indicates that calculations do not represent the process well and suggests that the oxidation of N-methyl-4-piperidones is a more complex process. Calculations of the BDEs for labile C-H bonds in the compounds did not show significant differences, suggesting that neither of the two series of compounds has a significant preference for proton-coupled electron transfer (PCET). It was proposed that irreversible behavior of the compounds is due to a potential dimerization by a Shono-type oxidation reaction, which is a common electrochemical reaction in tertiary heterocyclic amines. Future studies dealing with the potential mechanisms for the oxidation of these compounds need to be carried out to fully understand their electrochemical behavior.

## Figures and Tables

**Table 1:** Studied compounds.

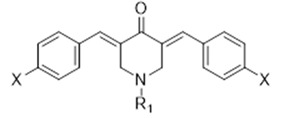		
Compound	R_1_	X
**C1**	CH_3_	H
**C2**	CH_3_	Br
**C3**	CH_3_	Cl
**C4**	CH_3_	CF_3_
**C5**	CH_3_	OCH_3_
**C6**	CH_2_Ph	H
**C7**	CH_2_Ph	Br
**C8**	CH_2_Ph	Cl
**C9**	CH_2_Ph	CF_3_
**C10**	CH_2_Ph	OCH_3_

**Table 2:** Synthesis of the obtained compounds.

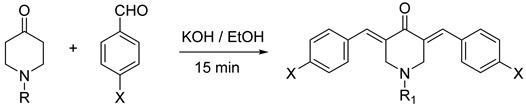		
Compound	% Yield	Ref.
**C1**	71	[1,3]
**C2**	62	[2]
**C3**	71	[1,3]
**C4**	72	[4]
**C5**	88	[1,3,4]
**C6**	81	[5]
**C7**	81	[5]
**C8**	50	[5]
**C9**	48	This work
**C10**	58	[5]

**Table 3:** Oxidation potentials of the compounds.

Compound	Eox (V)
**C1**	0.720
**C2**	0.770
**C3**	0.790
**C4**	0.770
**C5**	0.690
**C6**	0.830
**C7**	0.830
**C8**	0.840
**C9**	0.860
**C10**	0.730

**Table 4:** Number of electrons transferred (n) and surface concentration (Γ) for all the compounds.

Compound	n	Γ (mol/cm^2^)
**C1**	1.93	2.67 × 10^−7^
**C2**	1.77	3.55 × 10^−7^
**C3**	1.88	4.65 × 10^−7^
**C4**	2.19	1.72 × 10^−7^
**C5**	1.69	2.43 × 10^−7^
**C6**	1.68	5.01 × 10^−7^
**C7**	1.80	3.69 × 10^−7^
**C8**	1.78	2.76 × 10^−7^
**C9**	2.10	2.07 × 10^−7^
**C10**	1.87	4.89 × 10^−7^

**Table 5:** Experimental and theoretical oxidation potential (M06-2X) for all compounds, considering the oxidized species in the singlet state.

N-Substituent	Compound		Eox [V]			
		M06-2X (S)	M06-2X (T)	Exp	Error [V] (S)	Error [V] (T)
N-methyl	**C1**	2.26	1.96	0.72	1.54	1.24
	**C2**	2.07	2.02	0.77	1.3	1.25
	**C3**	2.05	1.98	0.79	1.26	1.19
	**C4**	2.44	2.32	0.77	1.67	1.55
	**C5**	1.60	1.45	0.69	0.91	0.76
				MAE	1.34	1.20
N-benzyl	**C6**	1.99	1.92	0.83	1.16	1.09
	**C7**	2.05	1.98	0.83	1.22	1.15
	**C8**	2.05	2.00	0.84	1.21	1.16
	**C9**	2.29	2.25	0.86	1.43	1.39
	**C10**	1.55	1.43	0.73	0.82	0.7
				MAE	1.17	1.10

**Table 6:** Experimental and theoretical oxidation potential (M06) for all compounds, considering the oxidized species in the singlet state.

N-Substituent	Compound		Eox [V]			
		M06 (S)	M06 (T)	Exp	Error [V] (S)	Error [V] (T)
N-methyl	**C1**	1.85	1.69	0.72	1.13	0.97
	**C2**	1.75	1.66	0.77	0.98	0.89
	**C3**	1.74	1.70	0.79	0.95	0.91
	**C4**	2.07	1.99	0.77	1.33	1.22
	**C5**	1.37	1.33	0.69	0.68	0.64
				MAE	1.01	0.93
N-benzyl	**C6**	1.57	1.58	0.83	0.74	0.75
	**C7**	1.62	1.58	0.83	0.79	0.75
	**C8**	1.67	1.55	0.84	0.83	0.71
	**C9**	1.76	1.82	0.86	0.90	0.96
	**C10**	1.21	1.23	0.73	0.48	0.50
				MAE	0.75	0.73

**Table 7:** Calculated HOMO and LUMO energies, as well as the HOMO–LUMO gap energy (GAP_H-L_). All values are in eV.

N-Substituent	Compound	E_HOMO_	E_LUMO_	GAP_H-L_
N-methyl	**C1**	−6.6844	−2.3063	4.3781
	**C2**	−6.7519	−2.5410	4.2109
	**C3**	−6.7651	−2.5264	4.2387
	**C4**	−7.0932	−2.8377	4.2555
	**C5**	−6.1293	−2.0417	4.0875
N-benzyl	**C6**	−6.6296	−2.2766	4.3529
	**C7**	−6.7279	−2.5091	4.2188
	**C8**	−6.7392	−2.4940	4.2452
	**C9**	−7.0073	−2.8015	4.2058
	**C10**	−6.1144	−2.0193	4.0951

**Table 8:** Calculated C–H bond dissociation enthalpies (BDEs).

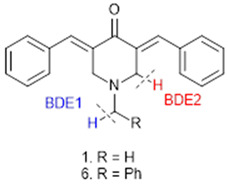		
Compound	BDE1 [kcal/mol]	BDE2 [kcal/mol]
**1**	83.47	61.50
**6**	71.20	60.15

## Data Availability

Not applicable.

## Supplementary Materials

The following supporting information can be downloaded at: https://www.mdpi.com/article/10.3390/ijms232315043/s1, Table S1: Optimized geometries for triplet dications of all compounds at the DFT M06/6-311+G(d,p) level; Table S2. Optimized geometries for singlet dications of all compounds at the DFT M06/6-311+G(d,p) level; Optimized geometry cartesian coordinates of all compounds (neutral, singlet dication, and triplet dication).
