# Supplementary material for: Study of the Electrochemical Behavior of N-Substituted-4-Piperidones Curcumin Analogs: A Combined Experimental and Theoretical Approach
Source: Int J Mol Sci. 2022 Nov 30;23(23):15043. doi: 10.3390/ijms232315043 (PMC9736124; doi:10.3390/ijms232315043)
Supplement: Supplementary file 1 [file ijms-23-15043-s001.zip › ijms-2017692-supplementary.pdf]

# Supplementary Material

# Study of the Electrochemical Behavior of N-Substituted-4-Piperidones Curcumin Analogs: A Combined Experimental and Theoretical Approach

**John Amalraj[1], Claudia E. Vergara[2], Matías Monroy-Cárdenas[1,3], Ramiro Araya-Maturana[1,3]* and Maximiliano Martínez-Cifuentes[4]***

[1] Instituto de Química de Recursos Naturales, Universidad de Talca, Talca 3460000, Chile
[2] Departamento de Ciencias Básicas, Facultad de Ciencias, Universidad Santo Tomás, Avda. Carlos Schorr 255, Talca, Chile
[3] MIBI: Interdisciplinary Group on Mitochondrial Targeting and Bioenergetics, Universidad de Talca, P.O. Box 747, Talca 3460000, Chile
[4] Departamento de Química Orgánica, Facultad de Ciencias Químicas, Universidad de Concepción, Edmundo Larenas 129, Concepción P.C. 4070371, Chile
* Correspondence: raraya@utalca.cl (R.A.-M.); maxmartinez@udec.cl (M.M.-C.)

Content

- Table S1. Optimized geometries for triplet dications of all compounds at DFT M06/6-311+G(d,p) level

- Table S2. Optimized geometries for singlet dications of all compounds at DFT M06/6-311+G(d,p) level

- Optimized geometry cartesian coordinates of all compounds (neutral, singlet dication and triplet dication)

**Table S1.** Optimized geometries for triplet dications of compounds 1 -10 at DFT M06/6-311+G(d,p) level

| | |
|---|---|
| 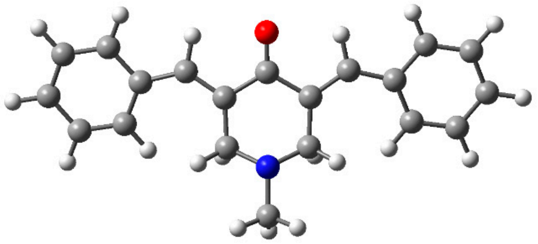<br>C1 | 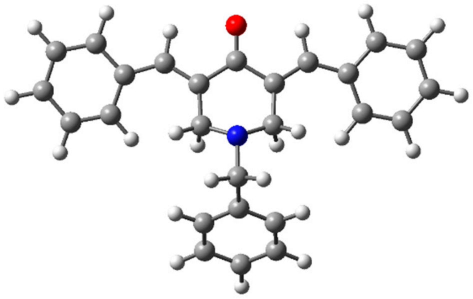<br>C6 |
| 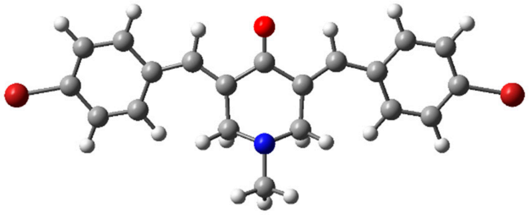<br>C2 | 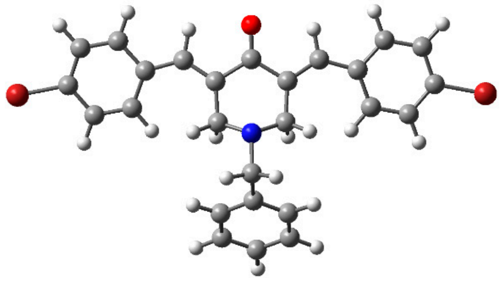<br>C7 |
| 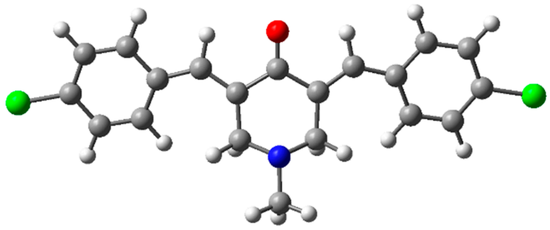<br>C3 | 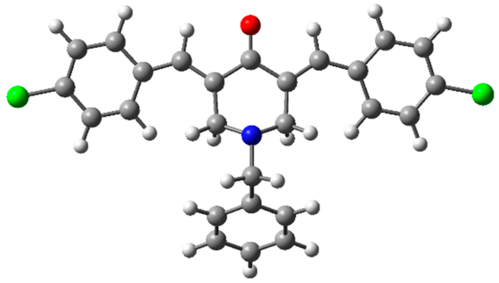<br>C8 |
| 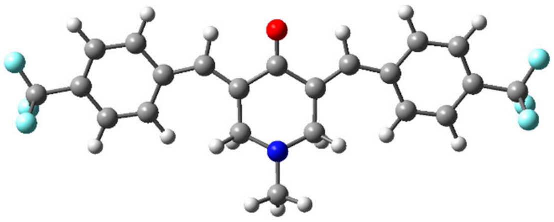<br>C4 | 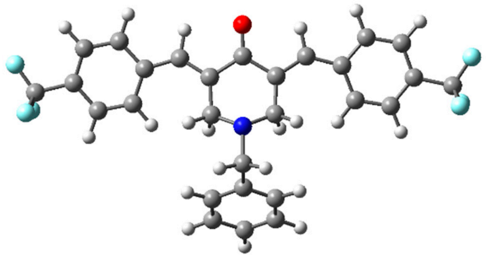<br>C9 |

| | |
|---|---|
| 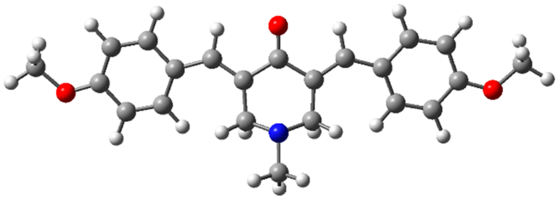<br>C5 | 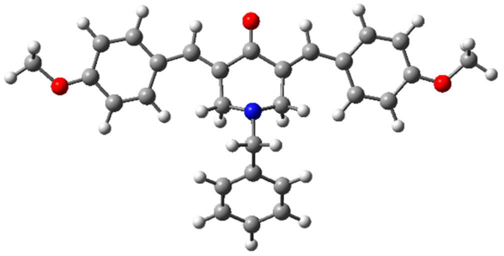<br>C10 |

**Table S2.** Optimized geometries for singlet dications of compounds 1 -10 at DFT M06/6-311+G(d,p) level

| | |
|---|---|
| 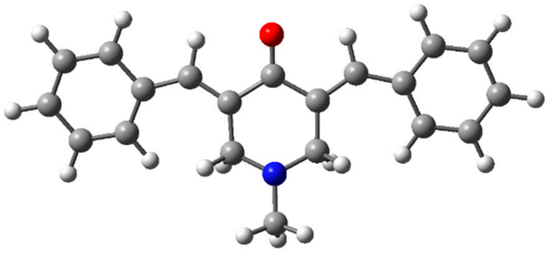<br>C1 | 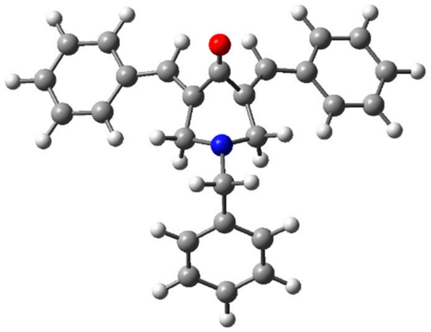<br>C6 |
| 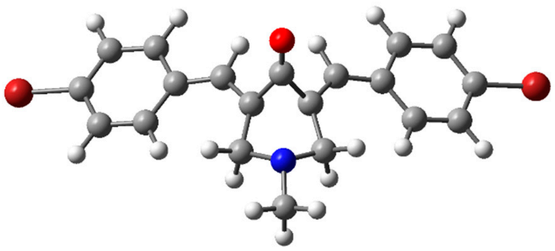<br>C2 | 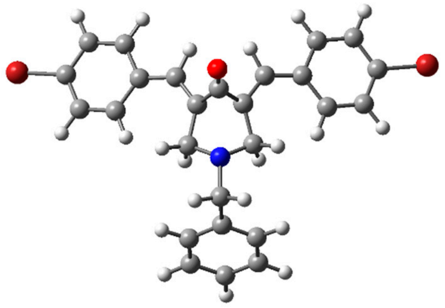<br>C7 |

| | |
|---|---|
| 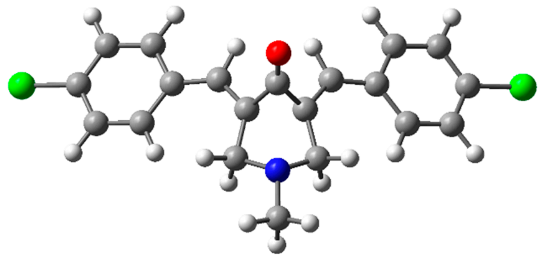 C3 | 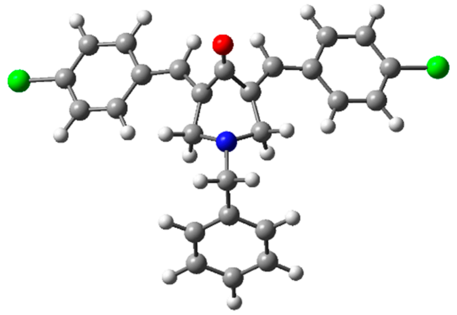 C8 |
| 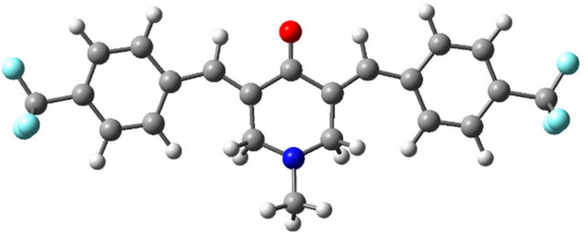 C4 | 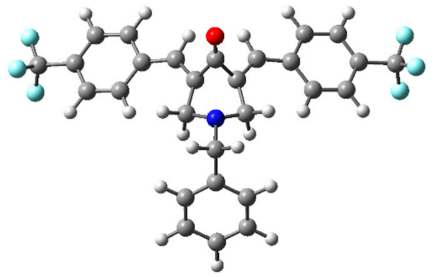 C9 |
| 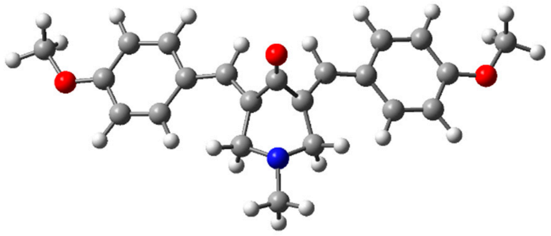 C5 | 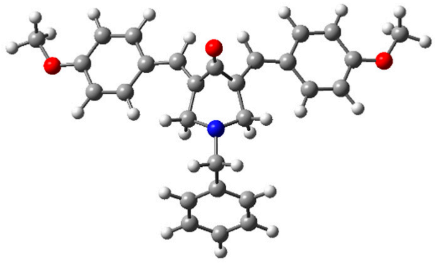 C10 |

- Optimized geometry cartesian coordinates of all compounds (neutral, singlet dication and triplet dication)

Neutral species at DFT M06/6-311+G(d,p) level

**C1**

0 1

| | | | |
|---|---|---|---|
| C | 1.18769500 | 1.18271000 | 0.07836400 |
| C | 0.00000100 | -1.09136500 | 0.03158700 |
| C | -1.18770100 | 1.18270600 | 0.07835900 |
| H | 1.19850000 | 1.63750400 | -0.94091100 |
| H | 2.06889300 | 1.57420900 | 0.60169100 |
| H | -1.19850200 | 1.63749700 | -0.94091700 |
| H | -2.06890400 | 1.57420300 | 0.60167900 |
| O | 0.00000400 | -2.30782900 | 0.05792300 |
| N | -0.00000600 | 1.60220900 | 0.79306900 |
| C | -0.00000900 | 3.03078500 | 1.00231700 |
| H | 0.88636000 | 3.32610200 | 1.57159400 |
| H | -0.00000800 | 3.59403600 | 0.04704800 |
| H | -0.88638200 | 3.32609900 | 1.57159000 |
| C | -1.27114800 | -0.31104700 | -0.00442400 |
| C | 1.27114700 | -0.31104200 | -0.00442200 |
| C | -2.40968700 | -1.02089400 | -0.07490100 |
| H | -2.27155500 | -2.10227600 | -0.04657400 |
| C | 2.40968800 | -1.02088700 | -0.07489900 |
| H | 2.27155700 | -2.10226900 | -0.04656600 |
| C | -3.79152600 | -0.56669700 | -0.13737600 |
| C | -4.78707100 | -1.42537700 | 0.34694200 |
| C | -4.18969600 | 0.66031600 | -0.68208400 |
| C | -6.12100500 | -1.05712800 | 0.33078900 |
| H | -4.49399800 | -2.39215100 | 0.74831400 |

| | | | |
|---|---|---|---|
| C | -5.52728700 | 1.02308200 | -0.71067400 |
| H | -3.45294200 | 1.32090600 | -1.12578300 |
| C | -6.49604300 | 0.17233100 | -0.19627500 |
| H | -6.87365000 | -1.73447400 | 0.72243600 |
| H | -5.81662700 | 1.97420600 | -1.14735000 |
| H | -7.54243700 | 0.46046400 | -0.21824400 |
| C | 3.79152800 | -0.56669200 | -0.13737400 |
| C | 4.78706900 | -1.42536900 | 0.34695400 |
| C | 4.18970300 | 0.66031400 | -0.68209600 |
| C | 6.12100500 | -1.05712400 | 0.33080100 |
| H | 4.49399300 | -2.39213800 | 0.74833600 |
| C | 5.52729500 | 1.02307600 | -0.71068700 |
| H | 3.45295300 | 1.32090000 | -1.12580600 |
| C | 6.49604700 | 0.17232900 | -0.19627600 |
| H | 6.87364700 | -1.73446700 | 0.72245700 |
| H | 5.81663900 | 1.97419400 | -1.14737300 |
| H | 7.54244200 | 0.46045900 | -0.21824500 |

C2

0 1

| | | | |
|---|---|---|---|
| C | 0.95964500 | 0.20247300 | 1.18748300 |
| C | -1.31177200 | 0.08139000 | 0.00000000 |
| C | 0.95964500 | 0.20247300 | -1.18748300 |
| H | 1.45086900 | -0.79967400 | 1.19857400 |
| H | 1.33187700 | 0.74069800 | 2.06801400 |
| H | 1.45086900 | -0.79967400 | -1.19857400 |
| H | 1.33187700 | 0.74069800 | -2.06801400 |
| O | -2.52805600 | 0.07049900 | 0.00000000 |
| N | 1.35240400 | 0.93194100 | 0.00000000 |

| | | | |
|---|---|---|---|
| C | 2.77199800 | 1.19740000 | 0.00000000 |
| H | 3.04437700 | 1.77819100 | 0.88613900 |
| H | 3.37287700 | 0.26551100 | 0.00000000 |
| H | 3.04437700 | 1.77819100 | -0.88613900 |
| C | -0.53028300 | 0.06817200 | -1.27095900 |
| C | -0.53028300 | 0.06817200 | 1.27095900 |
| C | -1.23745800 | -0.02810100 | -2.40904500 |
| H | -2.31936800 | -0.03310100 | -2.27271500 |
| C | -1.23745800 | -0.02810100 | 2.40904500 |
| H | -2.31936800 | -0.03310100 | 2.27271500 |
| C | -0.77748000 | -0.08202900 | -3.78857400 |
| C | -1.64153200 | 0.37788800 | -4.79058500 |
| C | 0.46262100 | -0.59896400 | -4.18171000 |
| C | -1.27426900 | 0.36852900 | -6.12404100 |
| H | -2.61927400 | 0.75896700 | -4.50771200 |
| C | 0.83709000 | -0.62904600 | -5.51516500 |
| H | 1.13363500 | -1.02430800 | -3.44360400 |
| C | -0.03015900 | -0.13450400 | -6.47755900 |
| H | -1.94946500 | 0.74011900 | -6.88726200 |
| H | 1.79497600 | -1.04473000 | -5.80905400 |
| C | -0.77748000 | -0.08202900 | 3.78857400 |
| C | -1.64153200 | 0.37788800 | 4.79058500 |
| C | 0.46262100 | -0.59896400 | 4.18171000 |
| C | -1.27426900 | 0.36852900 | 6.12404100 |
| H | -2.61927400 | 0.75896700 | 4.50771200 |
| C | 0.83709000 | -0.62904600 | 5.51516500 |
| H | 1.13363500 | -1.02430800 | 3.44360400 |
| C | -0.03015900 | -0.13450400 | 6.47755900 |
| H | -1.94946500 | 0.74011900 | 6.88726200 |

| | | | |
|---|---|---|---|
| H | 1.79497600 | -1.04473000 | 5.80905400 |
| Br | 0.48100100 | -0.16844500 | -8.30196000 |
| Br | 0.48100100 | -0.16844500 | 8.30196000 |

C3

0 1

| | | | |
|---|---|---|---|
| C | 1.07314600 | 0.13969300 | 1.18789700 |
| C | -1.19990700 | 0.05895300 | 0.00000000 |
| C | 1.07314600 | 0.13969300 | -1.18789700 |
| H | 1.54449000 | -0.87203900 | 1.20055200 |
| H | 1.45580000 | 0.67147600 | 2.06797800 |
| H | 1.54449000 | -0.87203900 | -1.20055200 |
| H | 1.45580000 | 0.67147600 | -2.06797800 |
| O | -2.41636300 | 0.06767000 | 0.00000000 |
| N | 1.48106400 | 0.86020100 | 0.00000000 |
| C | 2.90590500 | 1.09563400 | 0.00000000 |
| H | 3.19050900 | 1.67054000 | 0.88613400 |
| H | 3.48677000 | 0.15116300 | 0.00000000 |
| H | 3.19050900 | 1.67054000 | -0.88613400 |
| C | -0.41905500 | 0.03402700 | -1.27097400 |
| C | -0.41905500 | 0.03402700 | 1.27097400 |
| C | -1.12894700 | -0.04645400 | -2.40875200 |
| H | -2.21047900 | -0.03258600 | -2.27009100 |
| C | -1.12894700 | -0.04645400 | 2.40875200 |
| H | -2.21047900 | -0.03258600 | 2.27009100 |
| C | -0.67473500 | -0.10472400 | -3.78968600 |
| C | -1.53991100 | 0.36279600 | -4.78749000 |
| C | 0.55938700 | -0.63145800 | -4.18953400 |
| C | -1.17987800 | 0.35147000 | -6.12232700 |

| | | | |
|---|---|---|---|
| H | -2.51294700 | 0.75167600 | -4.49944800 |
| C | 0.92738800 | -0.66330000 | -5.52428600 |
| H | 1.23042200 | -1.06306700 | -3.45533900 |
| C | 0.05828300 | -0.16111600 | -6.48011900 |
| H | -1.85177000 | 0.72769500 | -6.88609400 |
| H | 1.87960500 | -1.08437300 | -5.82872800 |
| C | -0.67473500 | -0.10472400 | 3.78968600 |
| C | -1.53991100 | 0.36279600 | 4.78749000 |
| C | 0.55938700 | -0.63145800 | 4.18953400 |
| C | -1.17987800 | 0.35147000 | 6.12232700 |
| H | -2.51294700 | 0.75167600 | 4.49944800 |
| C | 0.92738800 | -0.66330000 | 5.52428600 |
| H | 1.23042200 | -1.06306700 | 3.45533900 |
| C | 0.05828300 | -0.16111600 | 6.48011900 |
| H | -1.85177000 | 0.72769500 | 6.88609400 |
| H | 1.87960500 | -1.08437300 | 5.82872800 |
| Cl | 0.52003800 | -0.19268600 | -8.16096300 |
| Cl | 0.52003800 | -0.19268600 | 8.16096300 |

C4

0 1

| | | | |
|---|---|---|---|
| C | 1.18681400 | -0.92930900 | -0.24560000 |
| C | 0.00000400 | 1.34124400 | -0.10445800 |
| C | -1.18685800 | -0.92928400 | -0.24554100 |
| H | 1.19417100 | -1.43303500 | 0.75003000 |
| H | 2.06770200 | -1.29631000 | -0.78635900 |
| H | -1.19416200 | -1.43298500 | 0.75010100 |
| H | -2.06778200 | -1.29628900 | -0.78623900 |
| O | 0.00002100 | 2.55623800 | -0.09070400 |

| | | | |
|---|---|---|---|
| N | -0.00004400 | -1.30813700 | -0.98303600 |
| C | -0.00006500 | -2.72309600 | -1.27537300 |
| H | 0.88591400 | -2.98432900 | -1.86124200 |
| H | -0.00004900 | -3.34103800 | -0.35502000 |
| H | -0.88607700 | -2.98431200 | -1.86120000 |
| C | -1.27209800 | 0.55875300 | -0.09118200 |
| C | 1.27208600 | 0.55872300 | -0.09120200 |
| C | -2.40797800 | 1.26500600 | 0.02480400 |
| H | -2.27455400 | 2.34702000 | 0.04342100 |
| C | 2.40797800 | 1.26495700 | 0.02480000 |
| H | 2.27456200 | 2.34697300 | 0.04342500 |
| C | -3.78634700 | 0.79737700 | 0.08198400 |
| C | -4.79288300 | 1.65296100 | -0.37820400 |
| C | -4.16456900 | -0.44698800 | 0.60378100 |
| C | -6.12334400 | 1.27134500 | -0.36388700 |
| H | -4.51676800 | 2.63119800 | -0.76182900 |
| C | -5.49284400 | -0.82699400 | 0.63398000 |
| H | -3.41748500 | -1.10847200 | 1.02750700 |
| C | -6.47286000 | 0.02730100 | 0.14058700 |
| H | -6.88984300 | 1.94154900 | -0.73700300 |
| H | -5.77556600 | -1.78897600 | 1.05172500 |
| C | 3.78634900 | 0.79733100 | 0.08197600 |
| C | 4.79287900 | 1.65294100 | -0.37817800 |
| C | 4.16458500 | -0.44704600 | 0.60373800 |
| C | 6.12334500 | 1.27134000 | -0.36387100 |
| H | 4.51675500 | 2.63118700 | -0.76177400 |
| C | 5.49286400 | -0.82703800 | 0.63392600 |
| H | 3.41751200 | -1.10855100 | 1.02744600 |
| C | 6.47287200 | 0.02728000 | 0.14055600 |

| | | | |
|---|---|---|---|
| H | 6.88983600 | 1.94156400 | -0.73696600 |
| H | 5.77559700 | -1.78903000 | 1.05164100 |
| C | 7.89904400 | -0.42362100 | 0.19185800 |
| C | -7.89902400 | -0.42363000 | 0.19186000 |
| F | 8.73985800 | 0.47007800 | -0.32966700 |
| F | 8.07261500 | -1.57225000 | -0.47735300 |
| F | 8.29421600 | -0.65481700 | 1.45210100 |
| F | -8.73987000 | 0.47015100 | -0.32947000 |
| F | -8.07259700 | -1.57213800 | -0.47756200 |
| F | -8.29414700 | -0.65507100 | 1.45207200 |

C5

0 1

| | | | |
|---|---|---|---|
| C | 1.18818500 | 1.18642600 | 0.12630200 |
| C | 0.00000900 | -1.08699500 | 0.09221500 |
| C | -1.18825900 | 1.18638200 | 0.12628100 |
| H | 1.20527400 | 1.64257400 | -0.89266100 |
| H | 2.06757600 | 1.57555200 | 0.65501200 |
| H | -1.20534100 | 1.64252100 | -0.89268600 |
| H | -2.06768000 | 1.57547600 | 0.65496500 |
| O | 0.00003400 | -2.30501800 | 0.13035800 |
| N | -0.00005200 | 1.61150200 | 0.83668200 |
| C | -0.00008100 | 3.04079500 | 1.03673600 |
| H | 0.88634900 | 3.33999600 | 1.60413500 |
| H | -0.00008200 | 3.59862200 | 0.07809200 |
| H | -0.88653300 | 3.33996300 | 1.60411800 |
| C | -1.26865600 | -0.30770400 | 0.04622600 |
| C | 1.26864400 | -0.30765800 | 0.04623200 |
| C | -2.40842300 | -1.01735300 | -0.03290100 |

| | | | |
|---|---|---|---|
| H | -2.26616300 | -2.09813200 | 0.00245700 |
| C | 2.40842900 | -1.01728500 | -0.03288100 |
| H | 2.26617900 | -2.09806300 | 0.00254800 |
| C | -3.78870000 | -0.57485900 | -0.12104300 |
| C | -4.79162500 | -1.45540200 | 0.29356100 |
| C | -4.19802600 | 0.66899400 | -0.63010900 |
| C | -6.13458600 | -1.11608800 | 0.25552100 |
| H | -4.50449100 | -2.43529700 | 0.66663600 |
| C | -5.53037300 | 1.01577300 | -0.68863300 |
| H | -3.46329300 | 1.36150500 | -1.02540800 |
| C | -6.51075900 | 0.13156200 | -0.23588200 |
| H | -6.87456700 | -1.82958700 | 0.59939800 |
| H | -5.84816700 | 1.97047400 | -1.09549700 |
| C | 3.78871300 | -0.57480500 | -0.12105400 |
| C | 4.79161300 | -1.45529800 | 0.29371700 |
| C | 4.19808100 | 0.66895400 | -0.63032300 |
| C | 6.13458000 | -1.11601200 | 0.25567000 |
| H | 4.50445200 | -2.43512800 | 0.66694200 |
| C | 5.53043600 | 1.01570000 | -0.68885900 |
| H | 3.46338200 | 1.36140300 | -1.02578500 |
| C | 6.51079200 | 0.13155100 | -0.23592100 |
| H | 6.87453900 | -1.82946800 | 0.59968900 |
| H | 5.84826000 | 1.97032400 | -1.09587900 |
| C | -8.81470700 | -0.29722100 | 0.09378700 |
| H | -8.72066300 | -0.54374700 | 1.15910700 |
| H | -9.75147100 | 0.23615000 | -0.06748600 |
| H | -8.82537500 | -1.22644200 | -0.49023900 |
| C | 8.81472000 | -0.29721600 | 0.09390200 |
| H | 8.82539200 | -1.22653600 | -0.48996800 |

| | | | |
|---|---|---|---|
| H | 9.75149900 | 0.23611100 | -0.06742800 |
| H | 8.72063400 | -0.54356200 | 1.15925900 |
| O | -7.78656100 | 0.56511600 | -0.32898100 |
| O | 7.78660400 | 0.56506700 | -0.32904800 |

- Singlet dication species at DFT M06/6-311+G(d,p) level

C1

2 1

| | | | |
|---|---|---|---|
| C | 1.24075200 | 1.34047000 | 0.13235100 |
| C | 0.00000200 | -0.88826000 | 0.12104800 |
| C | -1.24076000 | 1.34046500 | 0.13235600 |
| H | 1.57527900 | 1.70705500 | -0.87067700 |
| H | 2.00430500 | 1.74326500 | 0.81741200 |
| H | -1.57529800 | 1.70705300 | -0.87066700 |
| H | -2.00431100 | 1.74325100 | 0.81742500 |
| O | 0.00000500 | -2.09246100 | 0.21879400 |
| N | -0.00000500 | 1.97265400 | 0.41809700 |
| C | -0.00000800 | 3.41961700 | 0.53349800 |
| H | 0.88646200 | 3.74715200 | 1.08034900 |
| H | -0.00001300 | 3.90118200 | -0.45537900 |
| H | -0.88647800 | 3.74714700 | 1.08035400 |
| C | -1.29193200 | -0.13272300 | 0.07731800 |
| C | 1.29193100 | -0.13271800 | 0.07731800 |
| C | -2.42936500 | -0.89808400 | 0.01129400 |
| H | -2.21628000 | -1.96597700 | 0.07440600 |
| C | 2.42936700 | -0.89807700 | 0.01130100 |
| H | 2.21628300 | -1.96596900 | 0.07443000 |
| C | -3.81226700 | -0.57612700 | -0.09298000 |
| C | -4.72158400 | -1.64830300 | 0.14284500 |

| | | | |
|---|---|---|---|
| C | -4.34319400 | 0.69509600 | -0.41922500 |
| C | -6.08137800 | -1.44820000 | 0.10804000 |
| H | -4.32121600 | -2.63198700 | 0.37087700 |
| C | -5.70065400 | 0.88668100 | -0.46725500 |
| H | -3.69454800 | 1.52490100 | -0.67294700 |
| C | -6.57637000 | -0.18147400 | -0.19410300 |
| H | -6.76252100 | -2.26809500 | 0.30479800 |
| H | -6.10796800 | 1.85653200 | -0.73072200 |
| H | -7.64842600 | -0.01600600 | -0.23771100 |
| C | 3.81226900 | -0.57612300 | -0.09297900 |
| C | 4.72158500 | -1.64829000 | 0.14288800 |
| C | 4.34319900 | 0.69508900 | -0.41926900 |
| C | 6.08137900 | -1.44818900 | 0.10808300 |
| H | 4.32121500 | -2.63196600 | 0.37095300 |
| C | 5.70065900 | 0.88667100 | -0.46730100 |
| H | 3.69455500 | 1.52488300 | -0.67303200 |
| C | 6.57637300 | -0.18147500 | -0.19410400 |
| H | 6.76252100 | -2.26807800 | 0.30487300 |
| H | 6.10797500 | 1.85651100 | -0.73080500 |
| H | 7.64843000 | -0.01600900 | -0.23771400 |

C2

2 1

| | | | |
|---|---|---|---|
| C | 0.43045800 | 2.51849300 | 1.16066700 |
| C | -1.65755000 | 1.82917500 | 0.00000000 |
| C | 0.43045800 | 2.51849300 | -1.16066700 |
| H | 1.46746200 | 2.16175800 | 1.32386100 |
| H | 0.08717700 | 3.05722800 | 2.05074100 |
| H | 1.46746200 | 2.16175800 | -1.32386100 |

| | | | |
|---|---|---|---|
| H | 0.08717700 | 3.05722800 | -2.05074100 |
| O | -2.79878100 | 2.07118600 | 0.00000000 |
| N | 0.30273900 | 3.36979700 | 0.00000000 |
| C | 1.17010100 | 4.53410000 | 0.00000000 |
| H | 0.96704100 | 5.14280600 | 0.88341400 |
| H | 2.23519400 | 4.24904100 | 0.00000000 |
| H | 0.96704100 | 5.14280600 | -0.88341400 |
| C | -0.47795200 | 1.35996800 | -0.82478400 |
| C | -0.47795200 | 1.35996800 | 0.82478400 |
| C | -0.72177200 | 0.17278800 | -1.58345100 |
| H | -1.33360300 | -0.57777200 | -1.07940600 |
| C | -0.72177200 | 0.17278800 | 1.58345100 |
| H | -1.33360300 | -0.57777200 | 1.07940600 |
| C | -0.38693700 | -0.15138200 | -2.88695900 |
| C | -0.88838400 | -1.39265200 | -3.39651600 |
| C | 0.41057400 | 0.65586700 | -3.75768400 |
| C | -0.62537400 | -1.79711900 | -4.67251100 |
| H | -1.49897300 | -2.01800300 | -2.75107300 |
| C | 0.67758400 | 0.25123700 | -5.03150800 |
| H | 0.81945100 | 1.59832200 | -3.41684500 |
| C | 0.15970100 | -0.97523400 | -5.50066200 |
| H | -1.01297200 | -2.73498900 | -5.05457900 |
| H | 1.28322600 | 0.86078700 | -5.69293200 |
| C | -0.38693700 | -0.15138200 | 2.88695900 |
| C | -0.88838400 | -1.39265200 | 3.39651600 |
| C | 0.41057400 | 0.65586700 | 3.75768400 |
| C | -0.62537400 | -1.79711900 | 4.67251100 |
| H | -1.49897300 | -2.01800300 | 2.75107300 |
| C | 0.67758400 | 0.25123700 | 5.03150800 |

| | | | |
|---|---|---|---|
| H | 0.81945100 | 1.59832200 | 3.41684500 |
| C | 0.15970100 | -0.97523400 | 5.50066200 |
| H | -1.01297200 | -2.73498900 | 5.05457900 |
| H | 1.28322600 | 0.86078700 | 5.69293200 |
| Br | 0.52097400 | -1.50386600 | -7.22930800 |
| Br | 0.52097400 | -1.50386600 | 7.22930800 |

C3

2 1

| | | | |
|---|---|---|---|
| C | 2.24111900 | -0.36006200 | 1.16007000 |
| C | 1.39234000 | 1.66773400 | 0.00000000 |
| C | 2.24111900 | -0.36006200 | -1.16007000 |
| H | 1.96893100 | -1.42278400 | 1.31988100 |
| H | 2.75022800 | 0.02318900 | 2.05126400 |
| H | 1.96893100 | -1.42278400 | -1.31988100 |
| H | 2.75022800 | 0.02318900 | -2.05126400 |
| O | 1.54638600 | 2.82365800 | 0.00000000 |
| N | 3.07984200 | -0.16231600 | 0.00000000 |
| C | 4.31070100 | -0.93243600 | 0.00000000 |
| H | 4.90111400 | -0.68122100 | 0.88348500 |
| H | 4.11225200 | -2.01705400 | 0.00000000 |
| H | 4.90111400 | -0.68122100 | -0.88348500 |
| C | 1.01440700 | 0.45460800 | -0.82476500 |
| C | 1.01440700 | 0.45460800 | 0.82476500 |
| C | -0.18555400 | 0.60710600 | -1.58673200 |
| H | -0.98262900 | 1.16090700 | -1.08688100 |
| C | -0.18555400 | 0.60710600 | 1.58673200 |
| H | -0.98262900 | 1.16090700 | 1.08688100 |
| C | -0.47719300 | 0.24696600 | -2.89127800 |

| | | | |
|---|---|---|---|
| C | -1.74874800 | 0.65413500 | -3.41057800 |
| C | 0.39358500 | -0.49122400 | -3.75329600 |
| C | -2.12322200 | 0.35860200 | -4.68825500 |
| H | -2.42225300 | 1.21861700 | -2.77177600 |
| C | 0.02030600 | -0.79186800 | -5.02859600 |
| H | 1.35998700 | -0.82890200 | -3.40222000 |
| C | -1.23796200 | -0.36655700 | -5.50519800 |
| H | -3.08238400 | 0.67241600 | -5.08458200 |
| H | 0.67279200 | -1.35318600 | -5.68801900 |
| C | -0.47719300 | 0.24696600 | 2.89127800 |
| C | -1.74874800 | 0.65413500 | 3.41057800 |
| C | 0.39358500 | -0.49122400 | 3.75329600 |
| C | -2.12322200 | 0.35860200 | 4.68825500 |
| H | -2.42225300 | 1.21861700 | 2.77177600 |
| C | 0.02030600 | -0.79186800 | 5.02859600 |
| H | 1.35998700 | -0.82890200 | 3.40222000 |
| C | -1.23796200 | -0.36655700 | 5.50519800 |
| H | -3.08238400 | 0.67241600 | 5.08458200 |
| H | 0.67279200 | -1.35318600 | 5.68801900 |
| Cl | -1.68685100 | -0.74018600 | -7.08928600 |
| Cl | -1.68685100 | -0.74018600 | 7.08928600 |

C4

2 1

| | | | |
|---|---|---|---|
| C | -1.24232300 | 1.30062800 | -0.18657300 |
| C | 0.00000000 | -0.92770600 | -0.16358500 |
| C | 1.24233400 | 1.30062200 | -0.18655900 |
| H | -1.60696700 | 1.67288200 | 0.80371300 |
| H | -1.99015700 | 1.70026400 | -0.89273900 |

| | | | |
|---|---|---|---|
| H | 1.60696700 | 1.67287500 | 0.80373200 |
| H | 1.99017900 | 1.70025600 | -0.89271400 |
| O | -0.00000200 | -2.13312200 | -0.24176900 |
| N | 0.00000800 | 1.94155500 | -0.43722900 |
| C | 0.00000900 | 3.38979600 | -0.54545700 |
| H | -0.88707400 | 3.72089800 | -1.08915900 |
| H | -0.00003700 | 3.86296000 | 0.44734800 |
| H | 0.88713900 | 3.72090300 | -1.08907800 |
| C | 1.29141500 | -0.17137600 | -0.13008900 |
| C | -1.29141200 | -0.17137100 | -0.13009600 |
| C | 2.42915000 | -0.93763300 | -0.06862200 |
| H | 2.21300400 | -2.00547200 | -0.11901300 |
| C | -2.42915100 | -0.93762200 | -0.06862600 |
| H | -2.21300800 | -2.00546200 | -0.11900800 |
| C | 3.81522600 | -0.61892300 | 0.02040200 |
| C | 4.71433400 | -1.70265600 | -0.18520600 |
| C | 4.35700900 | 0.65734000 | 0.30197300 |
| C | 6.07925000 | -1.51677200 | -0.15898800 |
| H | 4.30872700 | -2.69083800 | -0.38184600 |
| C | 5.71430400 | 0.83920000 | 0.34123700 |
| H | 3.72005700 | 1.50426400 | 0.52519200 |
| C | 6.57866500 | -0.24799500 | 0.10222000 |
| H | 6.75613300 | -2.34615200 | -0.32921700 |
| H | 6.13831700 | 1.81218300 | 0.56997100 |
| C | -3.81522600 | -0.61891000 | 0.02039100 |
| C | -4.71433200 | -1.70265500 | -0.18517300 |
| C | -4.35701400 | 0.65736100 | 0.30191400 |
| C | -6.07924900 | -1.51677600 | -0.15895100 |
| H | -4.30872300 | -2.69084200 | -0.38178100 |

| | | | |
|---|---|---|---|
| C | -5.71431000 | 0.83921700 | 0.34118000 |
| H | -3.72006700 | 1.50429900 | 0.52509100 |
| C | -6.57866800 | -0.24799100 | 0.10221300 |
| H | -6.75612900 | -2.34616500 | -0.32914500 |
| H | -6.13832400 | 1.81220900 | 0.56987300 |
| C | -8.06820300 | 0.02130400 | 0.14436400 |
| C | 8.06819900 | 0.02130700 | 0.14436100 |
| F | -8.77228000 | -1.08531500 | 0.00262200 |
| F | -8.38369200 | 0.86354600 | -0.83482100 |
| F | -8.38423600 | 0.58979800 | 1.30218800 |
| F | 8.77228000 | -1.08530800 | 0.00261000 |
| F | 8.38367200 | 0.86355200 | -0.83482900 |
| F | 8.38424200 | 0.58980300 | 1.30218000 |

C5

2 1

| | | | |
|---|---|---|---|
| C | 1.15900800 | 2.33720100 | -0.34634700 |
| C | 0.00000000 | 1.44106700 | 1.67340000 |
| C | -1.15900600 | 2.33720100 | -0.34634900 |
| H | 1.32292000 | 2.06633800 | -1.40946800 |
| H | 2.05004100 | 2.84632200 | 0.03792900 |
| H | -1.32291700 | 2.06633900 | -1.40946900 |
| H | -2.05004000 | 2.84632400 | 0.03792700 |
| O | 0.00000000 | 1.54783200 | 2.83896100 |
| N | 0.00000100 | 3.17811000 | -0.15740800 |
| C | 0.00000200 | 4.39269100 | -0.95055500 |
| H | 0.88326200 | 4.98855200 | -0.71099200 |
| H | 0.00000300 | 4.17491300 | -2.03200200 |
| H | -0.88325800 | 4.98855300 | -0.71099400 |

| | | | |
|---|---|---|---|
| C | -0.82606900 | 1.10544300 | 0.46167600 |
| C | 0.82606900 | 1.10544200 | 0.46167700 |
| C | -1.59122200 | -0.10472600 | 0.57148400 |
| H | -1.09638600 | -0.91116500 | 1.11563200 |
| C | 1.59122100 | -0.10472700 | 0.57148300 |
| H | 1.09638400 | -0.91116800 | 1.11562900 |
| C | -2.88263000 | -0.38630000 | 0.18850700 |
| C | -3.41188900 | -1.66944200 | 0.55796800 |
| C | -3.75157900 | 0.50292800 | -0.53215000 |
| C | -4.68488600 | -2.04095700 | 0.26200800 |
| H | -2.77050400 | -2.35711400 | 1.10253900 |
| C | -5.01876000 | 0.14050600 | -0.83703600 |
| H | -3.39902700 | 1.47717100 | -0.84433600 |
| C | -5.51871100 | -1.13348200 | -0.44056900 |
| H | -5.05772800 | -3.01253600 | 0.56263900 |
| H | -5.69196800 | 0.79553700 | -1.37925700 |
| C | 2.88262900 | -0.38630100 | 0.18850600 |
| C | 3.41188700 | -1.66944400 | 0.55796500 |
| C | 3.75157900 | 0.50292800 | -0.53214800 |
| C | 4.68488500 | -2.04095900 | 0.26200600 |
| H | 2.77050100 | -2.35711800 | 1.10253400 |
| C | 5.01876000 | 0.14050700 | -0.83703300 |
| H | 3.39902700 | 1.47717200 | -0.84433100 |
| C | 5.51871100 | -1.13348200 | -0.44056800 |
| H | 5.05772700 | -3.01253900 | 0.56263500 |
| H | 5.69196900 | 0.79553900 | -1.37925200 |
| C | -7.40102800 | -2.60784400 | -0.42427100 |
| H | -7.42102800 | -2.73090700 | 0.66103600 |
| H | -8.41398600 | -2.51481200 | -0.80723200 |

| | | | |
|---|---|---|---|
| H | -6.89398200 | -3.44553500 | -0.90887900 |
| C | 7.40102700 | -2.60784500 | -0.42427200 |
| H | 6.89398100 | -3.44553400 | -0.90888200 |
| H | 8.41398500 | -2.51481300 | -0.80723300 |
| H | 7.42102700 | -2.73091100 | 0.66103500 |
| O | -6.74865000 | -1.37065500 | -0.76808400 |
| O | 6.74865000 | -1.37065500 | -0.76808300 |

C6

2 1

| | | | |
|---|---|---|---|
| C | 1.13690700 | 0.77539400 | 0.82365500 |
| C | 0.07378500 | -1.13061300 | 1.97824600 |
| C | -1.18069000 | 0.65320700 | 0.83357900 |
| H | 1.24710500 | 1.21566300 | -0.18478700 |
| H | 2.02717100 | 0.99593900 | 1.42341900 |
| H | -1.34849100 | 1.07998100 | -0.17286400 |
| H | -2.08214200 | 0.77916200 | 1.44382400 |
| O | 0.10201700 | -1.67529200 | 3.00645500 |
| N | -0.04629400 | 1.23419500 | 1.52365300 |
| C | -0.76552200 | -0.79545400 | 0.74882200 |
| C | 0.87652000 | -0.70796000 | 0.74845100 |
| C | -1.46580200 | -1.90319100 | 0.19440800 |
| H | -0.91459800 | -2.84551900 | 0.18257500 |
| C | 1.69342800 | -1.74539900 | 0.21999300 |
| H | 1.25579500 | -2.74544200 | 0.25543200 |
| C | -2.77702800 | -2.00753300 | -0.25703500 |
| C | -3.22068500 | -3.31062200 | -0.64014000 |
| C | -3.69081000 | -0.91695800 | -0.36130900 |
| C | -4.50103500 | -3.51280100 | -1.08696200 |

| | | | |
|---|---|---|---|
| H | -2.53013900 | -4.14605100 | -0.56444300 |
| C | -4.96601800 | -1.13226900 | -0.81112600 |
| H | -3.38394900 | 0.08724200 | -0.09840000 |
| C | -5.37336800 | -2.42521900 | -1.16967900 |
| H | -4.83660000 | -4.50346100 | -1.37143100 |
| H | -5.66441000 | -0.30729000 | -0.89369400 |
| C | 3.00320200 | -1.71445000 | -0.24685900 |
| C | 3.60268600 | -2.97342700 | -0.55896700 |
| C | 3.76993200 | -0.52638100 | -0.43649500 |
| C | 4.89341800 | -3.04202400 | -1.01654200 |
| H | 3.02428100 | -3.88235000 | -0.41775500 |
| C | 5.05457600 | -0.60854900 | -0.90256000 |
| H | 3.34048400 | 0.44638100 | -0.23303200 |
| C | 5.61860300 | -1.86068000 | -1.18710600 |
| H | 5.34868700 | -3.99908000 | -1.24398300 |
| H | 5.64054800 | 0.29073900 | -1.05467400 |
| C | -0.12334700 | 2.67799400 | 1.81414900 |
| H | 0.75786400 | 2.92520900 | 2.41593800 |
| H | -1.00039200 | 2.82351400 | 2.45375000 |
| C | -0.20177300 | 3.53370500 | 0.58238900 |
| C | -1.43978000 | 3.86431300 | 0.03221100 |
| C | 0.95799300 | 3.99741600 | -0.03737200 |
| C | -1.51756500 | 4.63041600 | -1.12185300 |
| H | -2.35389700 | 3.55786000 | 0.53898000 |
| C | 0.88288400 | 4.76447500 | -1.19143300 |
| H | 1.92828900 | 3.79576200 | 0.41450900 |
| C | -0.35528600 | 5.07578000 | -1.73773400 |
| H | -2.48580800 | 4.90267100 | -1.52933200 |
| H | 1.78986100 | 5.14136300 | -1.65297700 |

| | | | |
|---|---|---|---|
| H | -0.41514100 | 5.68841600 | -2.63114400 |
| H | -6.38875200 | -2.58209300 | -1.52242100 |
| H | 6.64081400 | -1.91067100 | -1.55107100 |

C7

2 1

| | | | |
|---|---|---|---|
| C | 1.16112300 | 1.49132800 | 1.07623800 |
| C | -0.00003200 | -0.19669700 | 2.46488700 |
| C | -1.16111800 | 1.49135700 | 1.07618600 |
| H | 1.31255600 | 1.76676000 | 0.01522100 |
| H | 2.05508100 | 1.75294100 | 1.65463600 |
| H | -1.31250700 | 1.76680800 | 0.01516700 |
| H | -2.05509100 | 1.75298200 | 1.65455500 |
| O | -0.00008700 | -0.60577000 | 3.55703900 |
| N | -0.00000200 | 2.11706200 | 1.67402500 |
| C | -0.82183300 | 0.02681400 | 1.20667400 |
| C | 0.82180000 | 0.02679300 | 1.20673200 |
| C | -1.58008800 | -1.11000200 | 0.79577700 |
| H | -1.07656000 | -2.07081900 | 0.91856100 |
| C | 1.58005000 | -1.11004000 | 0.79583200 |
| H | 1.07651000 | -2.07085000 | 0.91862600 |
| C | -2.89166500 | -1.20975500 | 0.35975300 |
| C | -3.41303300 | -2.52841900 | 0.16857800 |
| C | -3.75808300 | -0.10256800 | 0.10380500 |
| C | -4.70374300 | -2.73329100 | -0.22484700 |
| H | -2.76772800 | -3.38213800 | 0.35616400 |
| C | -5.04623000 | -0.30553900 | -0.29567200 |
| H | -3.39973600 | 0.91308200 | 0.21493800 |
| C | -5.53103800 | -1.62076600 | -0.45518800 |

| | | | |
|---|---|---|---|
| H | -5.09766100 | -3.73454400 | -0.35711800 |
| H | -5.70620300 | 0.53189800 | -0.49259100 |
| C | 2.89161500 | -1.20980800 | 0.35979500 |
| C | 3.41296200 | -2.52848100 | 0.16859900 |
| C | 3.75805200 | -0.10262900 | 0.10385400 |
| C | 4.70366300 | -2.73336800 | -0.22484000 |
| H | 2.76764400 | -3.38219000 | 0.35618000 |
| C | 5.04619000 | -0.30561700 | -0.29563500 |
| H | 3.39972500 | 0.91302500 | 0.21500800 |
| C | 5.53097600 | -1.62085300 | -0.45517400 |
| H | 5.09756400 | -3.73462600 | -0.35712800 |
| H | 5.70617800 | 0.53180900 | -0.49254600 |
| C | 0.00001500 | 3.58905300 | 1.72792600 |
| H | 0.88022700 | 3.88266500 | 2.31040800 |
| H | -0.88023900 | 3.88268600 | 2.31033300 |
| C | 0.00008100 | 4.24558000 | 0.37718300 |
| C | -1.20043100 | 4.54636400 | -0.26485300 |
| C | 1.20065800 | 4.54637700 | -0.26472800 |
| C | -1.20197400 | 5.11912300 | -1.52897500 |
| H | -2.14457400 | 4.37102000 | 0.24931700 |
| C | 1.20232900 | 5.11913500 | -1.52884900 |
| H | 2.14474700 | 4.37104300 | 0.24954400 |
| C | 0.00020900 | 5.39792800 | -2.16519100 |
| H | -2.14168500 | 5.37009500 | -2.01037000 |
| H | 2.14208800 | 5.37011700 | -2.01014500 |
| H | 0.00025800 | 5.85797200 | -3.14781900 |
| Br | -7.28162600 | -1.88173400 | -0.97817900 |
| Br | 7.28155100 | -1.88184200 | -0.97818200 |

C8

2 1

| | | | |
|---|---|---|---|
| C | 1.16080800 | 1.12407800 | 1.03789800 |
| C | -0.00003200 | -0.65168800 | 2.32054800 |
| C | -1.16082600 | 1.12410100 | 1.03788100 |
| H | 1.31346800 | 1.45924400 | -0.00584200 |
| H | 2.05391100 | 1.35250500 | 1.63149600 |
| H | -1.31346300 | 1.45926900 | -0.00586200 |
| H | -2.05393400 | 1.35254800 | 1.63146400 |
| O | -0.00004400 | -1.12550100 | 3.38594300 |
| N | -0.00000800 | 1.71692100 | 1.66803600 |
| C | -0.82324800 | -0.34606700 | 1.08185100 |
| C | 0.82319900 | -0.34608400 | 1.08186100 |
| C | -1.57998900 | -1.45224200 | 0.59133100 |
| H | -1.08022800 | -2.42074200 | 0.65523300 |
| C | 1.57992000 | -1.45227400 | 0.59133800 |
| H | 1.08013700 | -2.42076300 | 0.65523100 |
| C | -2.88551400 | -1.51748600 | 0.13172500 |
| C | -3.40523800 | -2.81661500 | -0.16979200 |
| C | -3.74529500 | -0.39078700 | -0.05463000 |
| C | -4.68566100 | -2.98664500 | -0.60897700 |
| H | -2.76554000 | -3.68421100 | -0.03419400 |
| C | -5.02385300 | -0.55731500 | -0.49669700 |
| H | -3.38936300 | 0.61195500 | 0.14417100 |
| C | -5.50299800 | -1.85506400 | -0.77103400 |
| H | -5.08306600 | -3.97083800 | -0.83029000 |
| H | -5.68364900 | 0.29054700 | -0.64267700 |
| C | 2.88544500 | -1.51754500 | 0.13173500 |
| C | 3.40513500 | -2.81668600 | -0.16979200 |

| | | | |
|---|---|---|---|
| C | 3.74525800 | -0.39086800 | -0.05460700 |
| C | 4.68555400 | -2.98674700 | -0.60897600 |
| H | 2.76541300 | -3.68426600 | -0.03420400 |
| C | 5.02381200 | -0.55742800 | -0.49667300 |
| H | 3.38935400 | 0.61188100 | 0.14420200 |
| C | 5.50292200 | -1.85518700 | -0.77102100 |
| H | 5.08293200 | -3.97094800 | -0.83029900 |
| H | 5.68363200 | 0.29041700 | -0.64264300 |
| C | 0.00001000 | 3.18565500 | 1.78822700 |
| H | 0.88034300 | 3.45318100 | 2.38281100 |
| H | -0.88035200 | 3.45321300 | 2.38275400 |
| C | 0.00007200 | 3.89918300 | 0.46627200 |
| C | -1.20044000 | 4.22299200 | -0.16472500 |
| C | 1.20064900 | 4.22294700 | -0.16462600 |
| C | -1.20192700 | 4.83741100 | -1.40924400 |
| H | -2.14461700 | 4.03076500 | 0.34337000 |
| C | 1.20226500 | 4.83736500 | -1.40914500 |
| H | 2.14477600 | 4.03067700 | 0.34354800 |
| C | 0.00020100 | 5.13627800 | -2.03632100 |
| H | -2.14156200 | 5.10502800 | -1.88175100 |
| H | 2.14194900 | 5.10494400 | -1.88157400 |
| H | 0.00025000 | 5.62810900 | -3.00345800 |
| Cl | -7.09329600 | -2.04864400 | -1.30976400 |
| Cl | 7.09321600 | -2.04880500 | -1.30975000 |

C9

2 1

| | | | |
|---|---|---|---|
| C | 1.16158400 | 1.46533800 | 0.98433900 |
| C | -0.00017000 | -0.16791000 | 2.39778800 |

| | | | |
|---|---|---|---|
| C | -1.16160000 | 1.46547100 | 0.98418700 |
| H | 1.29427600 | 1.76664000 | -0.07078500 |
| H | 2.05981000 | 1.71847100 | 1.55858200 |
| H | -1.29412100 | 1.76678500 | -0.07095700 |
| H | -2.05986600 | 1.71870700 | 1.55832000 |
| O | -0.00027200 | -0.55436000 | 3.49350900 |
| N | -0.00001500 | 2.06600400 | 1.61410100 |
| C | -0.81801300 | 0.00243100 | 1.10616900 |
| C | 0.81781200 | 0.00233900 | 1.10628200 |
| C | -1.57875000 | -1.14380700 | 0.76113400 |
| H | -1.07519100 | -2.10138000 | 0.90894000 |
| C | 1.57846100 | -1.14398100 | 0.76128300 |
| H | 1.07481500 | -2.10150800 | 0.90909300 |
| C | -2.90688900 | -1.25777100 | 0.35000600 |
| C | -3.42468000 | -2.58007700 | 0.23230400 |
| C | -3.75995000 | -0.15890700 | 0.04165600 |
| C | -4.72410800 | -2.79718200 | -0.15720600 |
| H | -2.78208500 | -3.42465300 | 0.46459000 |
| C | -5.05075300 | -0.38454700 | -0.35114900 |
| H | -3.39504100 | 0.85874400 | 0.09793100 |
| C | -5.53110400 | -1.69975300 | -0.44686700 |
| H | -5.12229100 | -3.80206900 | -0.24120800 |
| H | -5.70887700 | 0.44399000 | -0.59362400 |
| C | 2.90658800 | -1.25805700 | 0.35016300 |
| C | 3.42427000 | -2.58040800 | 0.23245400 |
| C | 3.75973300 | -0.15926100 | 0.04179300 |
| C | 4.72366800 | -2.79761900 | -0.15709800 |
| H | 2.78161300 | -3.42493100 | 0.46476000 |
| C | 5.05050100 | -0.38500800 | -0.35105900 |

| | | | |
|---|---|---|---|
| H | 3.39491200 | 0.85842100 | 0.09808300 |
| C | 5.53074000 | -1.70025400 | -0.44679700 |
| H | 5.12176900 | -3.80253700 | -0.24111100 |
| H | 5.70868500 | 0.44347600 | -0.59355800 |
| C | 0.00005500 | 3.53679800 | 1.74245300 |
| H | 0.88119100 | 3.80060400 | 2.33688700 |
| H | -0.88133700 | 3.80069400 | 2.33646500 |
| C | 0.00041800 | 4.24051900 | 0.41643900 |
| C | -1.20123900 | 4.55965000 | -0.21564500 |
| C | 1.20245200 | 4.55997000 | -0.21477700 |
| C | -1.20186500 | 5.16978400 | -1.46195400 |
| H | -2.14565500 | 4.37067700 | 0.29306200 |
| C | 1.20382800 | 5.17010400 | -1.46108400 |
| H | 2.14654400 | 4.37125200 | 0.29462400 |
| C | 0.00117100 | 5.46638800 | -2.08918300 |
| H | -2.14106400 | 5.43605800 | -1.93581600 |
| H | 2.14330100 | 5.43663300 | -1.93426000 |
| H | 0.00145700 | 5.95433700 | -3.05822900 |
| C | -6.96897000 | -1.88759200 | -0.89829700 |
| C | 6.96857000 | -1.88821300 | -0.89829500 |
| F | -7.31045000 | -3.16181700 | -0.92363400 |
| F | -7.10885600 | -1.37717000 | -2.11559400 |
| F | -7.77676000 | -1.23815200 | -0.07090100 |
| F | 7.30993900 | -3.16246600 | -0.92365100 |
| F | 7.77645200 | -1.23884400 | -0.07093500 |
| F | 7.10843900 | -1.37779900 | -2.11559600 |

C10

2 1

| | | | |
|---|---|---|---|
| C | 1.16072100 | 1.22032800 | 1.06946200 |
| C | 0.00011200 | -0.55585400 | 2.37748800 |
| C | -1.16068100 | 1.22022800 | 1.06949300 |
| H | 1.32539200 | 1.51449100 | 0.01468900 |
| H | 2.05106700 | 1.46889400 | 1.65986700 |
| H | -1.32540900 | 1.51439100 | 0.01472700 |
| H | -2.05103100 | 1.46871000 | 1.65992600 |
| O | 0.00014300 | -1.03513800 | 3.44512600 |
| N | 0.00000000 | 1.84448800 | 1.66459400 |
| C | -0.82486400 | -0.24918300 | 1.15518800 |
| C | 0.82503600 | -0.24911300 | 1.15517100 |
| C | -1.58265800 | -1.35659900 | 0.64862000 |
| H | -1.08334800 | -2.32481100 | 0.71761800 |
| C | 1.58291100 | -1.35646600 | 0.64858400 |
| H | 1.08367800 | -2.32471700 | 0.71758600 |
| C | -2.87493900 | -1.41615000 | 0.17715500 |
| C | -3.39734500 | -2.71369200 | -0.14475800 |
| C | -3.74938300 | -0.29027900 | 0.00140400 |
| C | -4.67152000 | -2.89421500 | -0.58223400 |
| H | -2.75019000 | -3.57783100 | -0.02126400 |
| C | -5.01865400 | -0.45901600 | -0.43628100 |
| H | -3.39954900 | 0.71128400 | 0.21504900 |
| C | -5.51240200 | -1.76215400 | -0.72998500 |
| H | -5.03836000 | -3.88789600 | -0.80898600 |
| H | -5.69766100 | 0.37533200 | -0.57397600 |
| C | 2.87519200 | -1.41592300 | 0.17710200 |
| C | 3.39768000 | -2.71342800 | -0.14482100 |

| | | | |
|---|---|---|---|
| C | 3.74956100 | -0.28999400 | 0.00134900 |
| C | 4.67186400 | -2.89386800 | -0.58230400 |
| H | 2.75058200 | -3.57761000 | -0.02132400 |
| C | 5.01884200 | -0.45864700 | -0.43634400 |
| H | 3.39966700 | 0.71154500 | 0.21500300 |
| C | 5.51267300 | -1.76175200 | -0.73005500 |
| H | 5.03876700 | -3.88752400 | -0.80906100 |
| H | 5.69779500 | 0.37574500 | -0.57403600 |
| C | -0.00010200 | 3.31518200 | 1.70680700 |
| H | 0.87976700 | 3.61437300 | 2.28735200 |
| H | -0.87997100 | 3.61417700 | 2.28745000 |
| C | -0.00029400 | 3.97006900 | 0.35427300 |
| C | -1.20017800 | 4.26920700 | -0.28951800 |
| C | 1.19940100 | 4.26989100 | -0.28956100 |
| C | -1.20232100 | 4.83675500 | -1.55608300 |
| H | -2.14432400 | 4.09374700 | 0.22460900 |
| C | 1.20117600 | 4.83744500 | -1.55612400 |
| H | 2.14366700 | 4.09500900 | 0.22454300 |
| C | -0.00066200 | 5.11341000 | -2.19392800 |
| H | -2.14243700 | 5.08458900 | -2.03851100 |
| H | 2.14113400 | 5.08583200 | -2.03857900 |
| H | -0.00080900 | 5.56904900 | -3.17867400 |
| O | -6.74500500 | -1.80918700 | -1.12702500 |
| O | 6.74527800 | -1.80870400 | -1.12709600 |
| C | -7.39154900 | -3.05423500 | -1.44897500 |
| H | -7.41179700 | -3.70425000 | -0.57100900 |
| H | -8.40521700 | -2.78661600 | -1.73578000 |
| H | -6.88188400 | -3.53664200 | -2.28642500 |
| C | 7.39190400 | -3.05370800 | -1.44905100 |

| | | | |
|---|---|---|---|
| H | 8.40555400 | -2.78602100 | -1.73585600 |
| H | 7.41219600 | -3.70372400 | -0.57108700 |
| H | 6.88227100 | -3.53614700 | -2.28650300 |

- Triplet dication species at DFT M06/6-311+G(d,p) level

C1

2 3

| | | | |
|---|---|---|---|
| C | 1.21703800 | 1.36489200 | 0.17203600 |
| C | -0.00000800 | -0.87357900 | 0.37211000 |
| C | -1.21699500 | 1.36492100 | 0.17202700 |
| H | 1.36557800 | 1.67668400 | -0.89269600 |
| H | 2.06069500 | 1.79998200 | 0.72298200 |
| H | -1.36551700 | 1.67671800 | -0.89270500 |
| H | -2.06064300 | 1.80003200 | 0.72296800 |
| O | -0.00002800 | -2.06431600 | 0.60171400 |
| N | 0.00002700 | 1.92720200 | 0.67939900 |
| C | 0.00004500 | 3.37602000 | 0.78171600 |
| H | 0.88345400 | 3.70436300 | 1.33329900 |
| H | 0.00005400 | 3.86236700 | -0.20697500 |
| H | -0.88335900 | 3.70438500 | 1.33329400 |
| C | -1.27464200 | -0.11670700 | 0.20728300 |
| C | 1.27464800 | -0.11673600 | 0.20729400 |
| C | -2.41485400 | -0.90150400 | 0.12345500 |
| H | -2.20871700 | -1.95819500 | 0.30176600 |
| C | 2.41484800 | -0.90154700 | 0.12345700 |
| H | 2.20870600 | -1.95824100 | 0.30174500 |
| C | -3.76977400 | -0.58433000 | -0.11063700 |
| C | -4.71167400 | -1.63727200 | 0.12272900 |
| C | -4.25876500 | 0.67382300 | -0.56713600 |

| | | | |
|---|---|---|---|
| C | -6.05689800 | -1.42846600 | -0.03425900 |
| H | -4.34051500 | -2.60456300 | 0.44896500 |
| C | -5.60138800 | 0.86942300 | -0.73665800 |
| H | -3.57424200 | 1.47345400 | -0.82066200 |
| C | -6.50741300 | -0.17492500 | -0.46117700 |
| H | -6.76649700 | -2.22405300 | 0.16189400 |
| H | -5.97735800 | 1.82123100 | -1.09503800 |
| H | -7.57165400 | -0.00469500 | -0.59701100 |
| C | 3.76976300 | -0.58436300 | -0.11063600 |
| C | 4.71167700 | -1.63730200 | 0.12268900 |
| C | 4.25873300 | 0.67381300 | -0.56709300 |
| C | 6.05689600 | -1.42847400 | -0.03430500 |
| H | 4.34053200 | -2.60460900 | 0.44889500 |
| C | 5.60135200 | 0.86943500 | -0.73662000 |
| H | 3.57419400 | 1.47344600 | -0.82057500 |
| C | 6.50739100 | -0.17491200 | -0.46118600 |
| H | 6.76650700 | -2.22405900 | 0.16181400 |
| H | 5.97730600 | 1.82126100 | -1.09496700 |
| H | 7.57162900 | -0.00466400 | -0.59702300 |

C2

2 3

| | | | |
|---|---|---|---|
| C | 1.29222700 | 0.38223600 | 1.20089300 |
| C | -0.95098400 | 0.57090800 | 0.00000000 |
| C | 1.29222700 | 0.38223600 | -1.20089300 |
| H | 1.64282700 | -0.67717000 | 1.27124500 |
| H | 1.72352500 | 0.90422500 | 2.06368700 |
| H | 1.64282700 | -0.67717000 | -1.27124500 |
| H | 1.72352500 | 0.90422500 | -2.06368700 |

| | | | |
|---|---|---|---|
| O | -2.14246100 | 0.79227300 | 0.00000000 |
| N | 1.77448900 | 1.01824100 | 0.00000000 |
| C | 3.22234600 | 1.15375000 | 0.00000000 |
| H | 3.53957700 | 1.71444800 | 0.88212100 |
| H | 3.73411100 | 0.17558400 | 0.00000000 |
| H | 3.53957700 | 1.71444800 | -0.88212100 |
| C | -0.19411100 | 0.40089300 | -1.27967000 |
| C | -0.19411100 | 0.40089300 | 1.27967000 |
| C | -0.97661100 | 0.30560300 | -2.40634200 |
| H | -2.03836800 | 0.45825400 | -2.20961700 |
| C | -0.97661100 | 0.30560300 | 2.40634200 |
| H | -2.03836800 | 0.45825400 | 2.20961700 |
| C | -0.64072400 | 0.08568800 | -3.76677800 |
| C | -1.67717100 | 0.30915500 | -4.72346400 |
| C | 0.62718500 | -0.35881600 | -4.23919500 |
| C | -1.45760200 | 0.15556000 | -6.06148200 |
| H | -2.65396500 | 0.62603100 | -4.37021500 |
| C | 0.85378700 | -0.52991900 | -5.57420500 |
| H | 1.41862100 | -0.60160300 | -3.54141300 |
| C | -0.18396900 | -0.26169800 | -6.49889300 |
| H | -2.24253400 | 0.34367000 | -6.78560900 |
| H | 1.81437700 | -0.87778700 | -5.93857700 |
| C | -0.64072400 | 0.08568800 | 3.76677800 |
| C | -1.67717100 | 0.30915500 | 4.72346400 |
| C | 0.62718500 | -0.35881600 | 4.23919500 |
| C | -1.45760200 | 0.15556000 | 6.06148200 |
| H | -2.65396500 | 0.62603100 | 4.37021500 |
| C | 0.85378700 | -0.52991900 | 5.57420500 |
| H | 1.41862100 | -0.60160300 | 3.54141300 |

| | | | |
|---|---|---|---|
| C | -0.18396900 | -0.26169800 | 6.49889300 |
| H | -2.24253400 | 0.34367000 | 6.78560900 |
| H | 1.81437700 | -0.87778700 | 5.93857700 |
| Br | 0.13188200 | -0.48048500 | -8.29631300 |
| Br | 0.13188200 | -0.48048500 | 8.29631300 |

C3

2 3

| | | | |
|---|---|---|---|
| C | 1.33333300 | 0.29452800 | 1.20337900 |
| C | -0.90496400 | 0.51288400 | 0.00000000 |
| C | 1.33333300 | 0.29452800 | -1.20337900 |
| H | 1.66135900 | -0.77176700 | 1.28708000 |
| H | 1.77176400 | 0.81419600 | 2.06418700 |
| H | 1.66135900 | -0.77176700 | -1.28708000 |
| H | 1.77176400 | 0.81419600 | -2.06418700 |
| O | -2.09388300 | 0.75200500 | 0.00000000 |
| N | 1.84001800 | 0.90209800 | 0.00000000 |
| C | 3.29080200 | 1.00478600 | 0.00000000 |
| H | 3.61963700 | 1.55873800 | 0.88202100 |
| H | 3.78107100 | 0.01624900 | 0.00000000 |
| H | 3.61963700 | 1.55873800 | -0.88202100 |
| C | -0.15134300 | 0.33352400 | -1.27818400 |
| C | -0.15134300 | 0.33352400 | 1.27818400 |
| C | -0.93757400 | 0.24697000 | -2.40928400 |
| H | -1.99548500 | 0.42315300 | -2.21050800 |
| C | -0.93757400 | 0.24697000 | 2.40928400 |
| H | -1.99548500 | 0.42315300 | 2.21050800 |
| C | -0.61006400 | 0.00851300 | -3.76378200 |
| C | -1.64912800 | 0.23262400 | -4.72125500 |

| | | | |
|---|---|---|---|
| C | 0.65271000 | -0.45505800 | -4.23736600 |
| C | -1.43577600 | 0.06327300 | -6.05743700 |
| H | -2.62122500 | 0.56363400 | -4.36789300 |
| C | 0.87167100 | -0.64244000 | -5.56973200 |
| H | 1.44473500 | -0.69934800 | -3.54088300 |
| C | -0.16798600 | -0.37200300 | -6.49272900 |
| H | -2.21667700 | 0.25046700 | -6.78605500 |
| H | 1.82405500 | -1.00532500 | -5.94061000 |
| C | -0.61006400 | 0.00851300 | 3.76378200 |
| C | -1.64912800 | 0.23262400 | 4.72125500 |
| C | 0.65271000 | -0.45505800 | 4.23736600 |
| C | -1.43577600 | 0.06327300 | 6.05743700 |
| H | -2.62122500 | 0.56363400 | 4.36789300 |
| C | 0.87167100 | -0.64244000 | 5.56973200 |
| H | 1.44473500 | -0.69934800 | 3.54088300 |
| C | -0.16798600 | -0.37200300 | 6.49272900 |
| H | -2.21667700 | 0.25046700 | 6.78605500 |
| H | 1.82405500 | -1.00532500 | 5.94061000 |
| Cl | 0.11554100 | -0.59528500 | -8.13935200 |
| Cl | 0.11554100 | -0.59528500 | 8.13935200 |

C4

2 3

| | | | |
|---|---|---|---|
| C | -1.23475200 | 1.31779200 | -0.29707300 |
| C | 0.00000100 | -0.92963600 | -0.41329000 |
| C | 1.23475000 | 1.31779300 | -0.29706100 |
| H | -1.47854100 | 1.66234600 | 0.73793000 |
| H | -2.03732600 | 1.72795600 | -0.92603400 |
| H | 1.47852900 | 1.66234900 | 0.73794400 |

| | | | |
|---|---|---|---|
| H | 2.03732900 | 1.72796000 | -0.92601100 |
| O | 0.00000000 | -2.12973400 | -0.57539900 |
| N | 0.00000000 | 1.91455300 | -0.69932800 |
| C | -0.00000100 | 3.35427800 | -0.87068300 |
| H | -0.88598700 | 3.65942100 | -1.43127100 |
| H | -0.00001500 | 3.87729000 | 0.09807700 |
| H | 0.88599700 | 3.65942500 | -1.43124900 |
| C | 1.27353500 | -0.16362300 | -0.29510300 |
| C | -1.27353500 | -0.16362400 | -0.29510900 |
| C | 2.41649000 | -0.94124400 | -0.21621300 |
| H | 2.20064300 | -2.00312300 | -0.34631100 |
| C | -2.41648900 | -0.94124500 | -0.21622100 |
| H | -2.20064100 | -2.00312500 | -0.34630700 |
| C | 3.78446800 | -0.62589700 | -0.03947100 |
| C | 4.70240800 | -1.69710400 | -0.26080100 |
| C | 4.30506200 | 0.64070200 | 0.34432400 |
| C | 6.05947100 | -1.50588000 | -0.15998900 |
| H | 4.31107700 | -2.67327100 | -0.53228100 |
| C | 5.65443700 | 0.82331900 | 0.46054800 |
| H | 3.64651400 | 1.46497000 | 0.58702800 |
| C | 6.53503700 | -0.24668200 | 0.19809000 |
| H | 6.75241000 | -2.31874000 | -0.34507100 |
| H | 6.06281900 | 1.78128000 | 0.76763800 |
| C | -3.78446700 | -0.62589700 | -0.03948300 |
| C | -4.70240700 | -1.69711100 | -0.26078200 |
| C | -4.30506200 | 0.64071400 | 0.34427200 |
| C | -6.05947000 | -1.50588800 | -0.15996500 |
| H | -4.31107600 | -2.67328400 | -0.53223900 |
| C | -5.65443800 | 0.82333200 | 0.46049500 |

| | | | |
|---|---|---|---|
| H | -3.64651800 | 1.46499600 | 0.58694000 |
| C | -6.53503800 | -0.24668100 | 0.19807900 |
| H | -6.75240800 | -2.31875400 | -0.34502100 |
| H | -6.06281800 | 1.78130400 | 0.76755100 |
| C | -8.02129900 | 0.02796400 | 0.32196800 |
| C | 8.02129900 | 0.02796500 | 0.32196400 |
| F | -8.73857900 | -1.06539700 | 0.15374200 |
| F | -8.36884600 | 0.92222500 | -0.59688600 |
| F | -8.27648500 | 0.53522000 | 1.52128700 |
| F | 8.73857600 | -1.06540600 | 0.15378500 |
| F | 8.36884000 | 0.92218100 | -0.59693800 |
| F | 8.27649600 | 0.53527900 | 1.52125500 |

C5

2 3

| | | | |
|---|---|---|---|
| C | 1.19699300 | 1.40858700 | 0.21592200 |
| C | -0.00000200 | -0.82577800 | 0.49737500 |
| C | -1.19698000 | 1.40859700 | 0.21592700 |
| H | 1.25042800 | 1.72632300 | -0.85391300 |
| H | 2.06271100 | 1.85978900 | 0.71574200 |
| H | -1.25041800 | 1.72633600 | -0.85390700 |
| H | -2.06269000 | 1.85980500 | 0.71575300 |
| O | -0.00000700 | -2.01175200 | 0.75615400 |
| N | 0.00001000 | 1.89735000 | 0.85807000 |
| C | 0.00001600 | 3.34703800 | 0.96285500 |
| H | 0.88244600 | 3.67716300 | 1.51586200 |
| H | 0.00001600 | 3.83790000 | -0.02668700 |
| H | -0.88240800 | 3.67717000 | 1.51586600 |
| C | -1.27773900 | -0.07760900 | 0.29800900 |

| | | | |
|---|---|---|---|
| C | 1.27774100 | -0.07761900 | 0.29800700 |
| C | -2.40953600 | -0.85941900 | 0.24089800 |
| H | -2.21957700 | -1.91334800 | 0.44631400 |
| C | 2.40953400 | -0.85943300 | 0.24089400 |
| H | 2.21957200 | -1.91336300 | 0.44630500 |
| C | -3.76003700 | -0.52228900 | -0.00510600 |
| C | -4.73498900 | -1.54178200 | 0.24515900 |
| C | -4.22342900 | 0.73266100 | -0.50352300 |
| C | -6.06781800 | -1.32927500 | 0.07280600 |
| H | -4.38889200 | -2.50785100 | 0.60107500 |
| C | -5.54794900 | 0.95811900 | -0.69398100 |
| H | -3.51783800 | 1.50932300 | -0.76903500 |
| C | -6.50196500 | -0.06273200 | -0.39836700 |
| H | -6.78259000 | -2.11505300 | 0.28686900 |
| H | -5.92585500 | 1.89811800 | -1.08182200 |
| C | 3.76003400 | -0.52230200 | -0.00510800 |
| C | 4.73498800 | -1.54179500 | 0.24514500 |
| C | 4.22342200 | 0.73265600 | -0.50350800 |
| C | 6.06781700 | -1.32928200 | 0.07279300 |
| H | 4.38889400 | -2.50787000 | 0.60104900 |
| C | 5.54794100 | 0.95812000 | -0.69396400 |
| H | 3.51782700 | 1.50932100 | -0.76900600 |
| C | 6.50196000 | -0.06273200 | -0.39836500 |
| H | 6.78259100 | -2.11506200 | 0.28684500 |
| H | 5.92584500 | 1.89812500 | -1.08179200 |
| C | -8.82044900 | -0.65957400 | -0.37414100 |
| H | -8.84989700 | -0.93790900 | 0.68179700 |
| H | -9.72311400 | -0.11751600 | -0.64351300 |
| H | -8.70437500 | -1.53850200 | -1.01222000 |

| | | | |
|---|---|---|---|
| C | 8.82044600 | -0.65956900 | -0.37414900 |
| H | 8.70437400 | -1.53848800 | -1.01224100 |
| H | 9.72310900 | -0.11750500 | -0.64351200 |
| H | 8.84989400 | -0.93791900 | 0.68178500 |
| O | -7.73807300 | 0.26287600 | -0.60855300 |
| O | 7.73806700 | 0.26288200 | -0.60854700 |

C6

2 3

| | | | |
|---|---|---|---|
| C | 1.22467300 | 0.19179500 | 0.65222500 |
| C | 0.04216800 | -2.00085300 | 0.09353300 |
| C | -1.19636400 | 0.15492200 | 0.67408300 |
| H | 1.32573800 | 0.84741300 | -0.25532700 |
| H | 2.08050800 | 0.44827100 | 1.28974300 |
| H | -1.33789800 | 0.81600300 | -0.22380500 |
| H | -2.04870200 | 0.37601500 | 1.32972000 |
| O | 0.05745200 | -3.19657000 | -0.10600000 |
| N | 0.01460100 | 0.53031100 | 1.34533500 |
| C | -1.23929900 | -1.24854800 | 0.21202100 |
| C | 1.30448500 | -1.21428600 | 0.20172800 |
| C | -2.37750300 | -1.97259400 | -0.10663600 |
| H | -2.16933500 | -3.02773800 | -0.29055500 |
| C | 2.45902200 | -1.91240300 | -0.11509100 |
| H | 2.27487200 | -2.97296000 | -0.29351100 |
| C | -3.73550300 | -1.59881000 | -0.19980900 |
| C | -4.67976700 | -2.66826500 | -0.28897000 |
| C | -4.22247000 | -0.26087100 | -0.22798900 |
| C | -6.02729700 | -2.41802800 | -0.33860600 |
| H | -4.31070100 | -3.68978900 | -0.29450800 |

| | | | |
|---|---|---|---|
| C | -5.56763900 | -0.01995500 | -0.29719200 |
| H | -3.53423100 | 0.57625000 | -0.23749900 |
| C | -6.47638200 | -1.09466300 | -0.33953300 |
| H | -6.73873800 | -3.23454100 | -0.38506500 |
| H | -5.94249100 | 0.99685800 | -0.33193400 |
| C | 3.80869500 | -1.50989200 | -0.20732300 |
| C | 4.77472300 | -2.56073900 | -0.28727000 |
| C | 4.26876800 | -0.16255300 | -0.23837800 |
| C | 6.11731200 | -2.28425200 | -0.32912900 |
| H | 4.42596700 | -3.58935100 | -0.29131500 |
| C | 5.60940500 | 0.10463800 | -0.29891000 |
| H | 3.56357400 | 0.66029900 | -0.25589800 |
| C | 6.53978400 | -0.95221200 | -0.33081400 |
| H | 6.84512900 | -3.08657900 | -0.36829600 |
| H | 5.96436200 | 1.12852700 | -0.33424500 |
| C | -0.00414400 | 1.91012300 | 1.84818700 |
| H | 0.89006300 | 2.04126400 | 2.46735000 |
| H | -0.86922300 | 1.99922800 | 2.51483400 |
| C | -0.05997900 | 2.96182000 | 0.76820000 |
| C | -1.28966800 | 3.43578300 | 0.30299300 |
| C | 1.11521200 | 3.46858000 | 0.20707000 |
| C | -1.34416700 | 4.36785700 | -0.72284500 |
| H | -2.20999000 | 3.10481300 | 0.78194800 |
| C | 1.06156400 | 4.40005600 | -0.81954200 |
| H | 2.08010600 | 3.16491500 | 0.61066500 |
| C | -0.16838100 | 4.84708100 | -1.28806700 |
| H | -2.30242700 | 4.74222400 | -1.06753200 |
| H | 1.97882800 | 4.79920600 | -1.23971300 |
| H | -0.21006800 | 5.59129500 | -2.07624800 |

| | | | |
|---|---|---|---|
| H | 7.60097700 | -0.72488300 | -0.37269500 |
| H | -7.54162200 | -0.88874600 | -0.38907500 |

C7

2 3

| | | | |
|---|---|---|---|
| C | 1.21466100 | 0.39872300 | 0.69397200 |
| C | 0.02429600 | -1.82839600 | 0.32297900 |
| C | -1.18790000 | 0.38232900 | 0.71317900 |
| H | 1.27899000 | 0.99494400 | -0.25106500 |
| H | 2.08023000 | 0.69423400 | 1.29979800 |
| H | -1.27677300 | 0.98459700 | -0.22599000 |
| H | -2.04826200 | 0.66224300 | 1.33387800 |
| O | 0.03034600 | -3.03666000 | 0.22884600 |
| N | 0.01676200 | 0.71387200 | 1.43311800 |
| C | -1.25598500 | -1.05819300 | 0.36307300 |
| C | 1.29718900 | -1.04431200 | 0.35361700 |
| C | -2.38520100 | -1.79412500 | 0.08619800 |
| H | -2.18888200 | -2.86066000 | -0.02797000 |
| C | 2.43199600 | -1.77253900 | 0.08017700 |
| H | 2.24358200 | -2.84128600 | -0.02650700 |
| C | -3.74520700 | -1.41450200 | -0.03747800 |
| C | -4.70037600 | -2.47411300 | -0.06604600 |
| C | -4.22038000 | -0.07764300 | -0.15991200 |
| C | -6.04021900 | -2.22557100 | -0.15226300 |
| H | -4.34554400 | -3.49822500 | 0.00177300 |
| C | -5.55748800 | 0.18013500 | -0.26495100 |
| H | -3.52406000 | 0.75150700 | -0.21067300 |
| C | -6.47959900 | -0.89182100 | -0.24895200 |
| H | -6.76284500 | -3.03384900 | -0.15313200 |

| | | | |
|---|---|---|---|
| H | -5.92468200 | 1.19514000 | -0.37155200 |
| C | 3.78970200 | -1.38502100 | -0.04458300 |
| C | 4.75125100 | -2.43919000 | -0.06130100 |
| C | 4.25711400 | -0.04638200 | -0.17678500 |
| C | 6.08983600 | -2.18337500 | -0.14426000 |
| H | 4.40218900 | -3.46478900 | 0.01353500 |
| C | 5.59300000 | 0.21873000 | -0.27840300 |
| H | 3.55531800 | 0.77743600 | -0.23752700 |
| C | 6.52157500 | -0.84764600 | -0.24953000 |
| H | 6.81732300 | -2.98724900 | -0.13568300 |
| H | 5.95459600 | 1.23503900 | -0.39177800 |
| C | 0.00685900 | 2.09563000 | 1.94151500 |
| H | 0.90397500 | 2.21711700 | 2.55862000 |
| H | -0.85321000 | 2.18202700 | 2.61517000 |
| C | -0.05102800 | 3.15726900 | 0.87572200 |
| C | -1.27991600 | 3.64607700 | 0.42315800 |
| C | 1.12088000 | 3.66627900 | 0.30888500 |
| C | -1.33804300 | 4.59869200 | -0.58200100 |
| H | -2.19862600 | 3.30497000 | 0.89830300 |
| C | 1.06651500 | 4.61864600 | -0.69699500 |
| H | 2.08667700 | 3.34263200 | 0.69414800 |
| C | -0.16398300 | 5.08236000 | -1.14804900 |
| H | -2.29671500 | 4.98496000 | -0.91242000 |
| H | 1.98294000 | 5.01994300 | -1.11718400 |
| H | -0.20742000 | 5.83935400 | -1.92393000 |
| Br | -8.28170700 | -0.53529300 | -0.37972400 |
| Br | 8.32181600 | -0.48116800 | -0.37451000 |

C8

2 3

| | | | |
|---|---|---|---|
| C | 1.20342100 | 0.29318200 | 0.67041400 |
| C | 0.00009200 | -1.91117100 | 0.22046300 |
| C | -1.20333000 | 0.29312000 | 0.67045900 |
| H | 1.29112200 | 0.91427000 | -0.25849300 |
| H | 2.06481100 | 0.56367600 | 1.29400600 |
| H | -1.29110200 | 0.91422300 | -0.25843100 |
| H | -2.06471300 | 0.56355500 | 1.29408800 |
| O | 0.00011700 | -3.11635700 | 0.08731100 |
| N | 0.00005000 | 0.62164700 | 1.39029900 |
| C | -1.27489000 | -1.13637700 | 0.28397100 |
| C | 1.27504300 | -1.13632100 | 0.28395700 |
| C | -2.41244800 | -1.86124400 | -0.00887200 |
| H | -2.21958000 | -2.92599800 | -0.14509300 |
| C | 2.41263000 | -1.86115100 | -0.00886000 |
| H | 2.21980200 | -2.92591400 | -0.14506900 |
| C | -3.76716300 | -1.47387600 | -0.12353400 |
| C | -4.72916600 | -2.52893200 | -0.17688100 |
| C | -4.23742200 | -0.13116700 | -0.21038800 |
| C | -6.06786100 | -2.27185300 | -0.24710400 |
| H | -4.37907500 | -3.55622500 | -0.13899400 |
| C | -5.57255800 | 0.13496900 | -0.29893200 |
| H | -3.53791900 | 0.69587100 | -0.24630400 |
| C | -6.49879200 | -0.93382300 | -0.30271800 |
| H | -6.79950200 | -3.07188400 | -0.26611900 |
| H | -5.94254800 | 1.15123500 | -0.37764200 |
| C | 3.76733600 | -1.47374000 | -0.12349100 |
| C | 4.72937300 | -2.52876700 | -0.17680000 |

| | | | |
|---|---|---|---|
| C | 4.23755500 | -0.13101600 | -0.21033400 |
| C | 6.06806300 | -2.27164600 | -0.24697600 |
| H | 4.37931300 | -3.55607000 | -0.13891900 |
| C | 5.57268500 | 0.13516100 | -0.29883100 |
| H | 3.53802500 | 0.69599900 | -0.24627600 |
| C | 6.49895300 | -0.93360200 | -0.30258000 |
| H | 6.79973000 | -3.07165400 | -0.26596100 |
| H | 5.94264600 | 1.15143800 | -0.37752900 |
| C | 0.00001500 | 2.00436800 | 1.89417500 |
| H | 0.87929300 | 2.11058500 | 2.53935100 |
| H | -0.87915100 | 2.11047600 | 2.53952200 |
| C | -0.00016400 | 3.06421900 | 0.82374700 |
| C | -1.20247300 | 3.56335400 | 0.31404200 |
| C | 1.20196800 | 3.56343000 | 0.31369900 |
| C | -1.20422700 | 4.51385300 | -0.69505200 |
| H | -2.14585000 | 3.23435800 | 0.74737400 |
| C | 1.20336900 | 4.51392800 | -0.69539800 |
| H | 2.14549100 | 3.23449600 | 0.74675900 |
| C | -0.00051700 | 4.98701200 | -1.20492800 |
| H | -2.14290300 | 4.90724600 | -1.07115800 |
| H | 2.14191300 | 4.90737900 | -1.07177400 |
| H | -0.00065300 | 5.74392400 | -1.98206300 |
| Cl | -8.15041800 | -0.59140400 | -0.39602000 |
| Cl | 8.15057100 | -0.59113200 | -0.39582400 |

C9

2 3

| | | | |
|---|---|---|---|
| C | 1.20241800 | 0.30720300 | 0.77708700 |
| C | -0.02225400 | -1.85409100 | 0.17110100 |

| | | | |
|---|---|---|---|
| C | -1.22889600 | 0.32393800 | 0.74987800 |
| H | 1.36175500 | 0.98136100 | -0.11014300 |
| H | 2.04312900 | 0.52553900 | 1.44932000 |
| H | -1.35180900 | 0.98537600 | -0.15210500 |
| H | -2.08087100 | 0.56793800 | 1.39799900 |
| O | -0.02725500 | -3.04556200 | -0.04522500 |
| N | -0.01765900 | 0.69815900 | 1.41654100 |
| C | -1.29122400 | -1.07915800 | 0.29088300 |
| C | 1.25189500 | -1.09092700 | 0.30266500 |
| C | -2.44047200 | -1.78407600 | -0.02505500 |
| H | -2.24609200 | -2.84098300 | -0.21493700 |
| C | 2.39677600 | -1.80195600 | -0.01510700 |
| H | 2.19572300 | -2.85574900 | -0.21480700 |
| C | -3.79724700 | -1.39403300 | -0.10611000 |
| C | -4.75047800 | -2.45232200 | -0.18397800 |
| C | -4.27119200 | -0.05341300 | -0.12846400 |
| C | -6.10019600 | -2.19319800 | -0.21704800 |
| H | -4.39415100 | -3.47823500 | -0.19479200 |
| C | -5.61327700 | 0.20041300 | -0.18133500 |
| H | -3.57828000 | 0.77945300 | -0.14714600 |
| C | -6.53061700 | -0.86909700 | -0.21216800 |
| H | -6.82342900 | -2.99995400 | -0.25627500 |
| H | -5.98636400 | 1.21940400 | -0.21237600 |
| C | 3.75635000 | -1.41990600 | -0.09302900 |
| C | 4.70289900 | -2.48255600 | -0.18508400 |
| C | 4.23832800 | -0.08217100 | -0.10313100 |
| C | 6.05404300 | -2.23086500 | -0.22274300 |
| H | 4.34075400 | -3.50631200 | -0.20424700 |
| C | 5.58166600 | 0.16446900 | -0.16110600 |

| | | | |
|---|---|---|---|
| H | 3.55083100 | 0.75525000 | -0.10885700 |
| C | 6.49247200 | -0.90953300 | -0.20819400 |
| H | 6.77198100 | -3.04166200 | -0.27344700 |
| H | 5.96004900 | 1.18172600 | -0.18399300 |
| C | -0.01118800 | 2.07923700 | 1.90893800 |
| H | 0.85070200 | 2.18254600 | 2.57764900 |
| H | -0.91034100 | 2.21229900 | 2.52025600 |
| C | 0.04356300 | 3.11869400 | 0.81437600 |
| C | -1.13265700 | 3.61645200 | 0.24922900 |
| C | 1.27454600 | 3.58485700 | 0.34357700 |
| C | -1.07840500 | 4.53063000 | -0.79364200 |
| H | -2.09748800 | 3.32126800 | 0.65917700 |
| C | 1.32824000 | 4.49920700 | -0.69894500 |
| H | 2.19477400 | 3.26590100 | 0.83075800 |
| C | 0.15197200 | 4.96939100 | -1.27019200 |
| H | -1.99571600 | 4.92273000 | -1.22021900 |
| H | 2.28630400 | 4.86812500 | -1.04980600 |
| H | 0.19291400 | 5.70181100 | -2.06936200 |
| C | -8.00558400 | -0.52254600 | -0.25008000 |
| C | 7.96893200 | -0.57078400 | -0.25480300 |
| F | 8.71894000 | -1.65137600 | -0.35232600 |
| F | 8.29907100 | 0.08686600 | 0.85145600 |
| F | 8.20649100 | 0.21706600 | -1.29742800 |
| F | -8.76153000 | -1.59950100 | -0.34089600 |
| F | -8.32434300 | 0.13816900 | 0.85768300 |
| F | -8.24587500 | 0.26504200 | -1.29201800 |

C10

2 3

| | | | |
|---|---|---|---|
| C | 1.19909700 | 0.38949300 | 0.58599000 |
| C | 0.00009100 | -1.83501400 | 0.23639600 |
| C | -1.19901200 | 0.38942900 | 0.58604600 |
| H | 1.26955500 | 0.97948000 | -0.35984100 |
| H | 2.06165500 | 0.68393500 | 1.19654000 |
| H | -1.26954600 | 0.97942500 | -0.35977300 |
| H | -2.06156000 | 0.68381800 | 1.19663700 |
| O | 0.00011900 | -3.04580600 | 0.14491000 |
| N | 0.00005100 | 0.69878400 | 1.32811000 |
| C | -1.27569100 | -1.06028800 | 0.26470500 |
| C | 1.27583900 | -1.06022700 | 0.26466900 |
| C | -2.41263100 | -1.79497300 | 0.00809400 |
| H | -2.22615500 | -2.86521200 | -0.08396400 |
| C | 2.41280500 | -1.79487200 | 0.00806300 |
| H | 2.22636700 | -2.86512000 | -0.08397500 |
| C | -3.76382200 | -1.40294300 | -0.11679000 |
| C | -4.73599300 | -2.45373300 | -0.13381100 |
| C | -4.23473200 | -0.06079800 | -0.24747300 |
| C | -6.07245700 | -2.20664600 | -0.20717200 |
| H | -4.38532200 | -3.47941600 | -0.06519900 |
| C | -5.56348700 | 0.20181900 | -0.33755400 |
| H | -3.53328700 | 0.76267700 | -0.31247800 |
| C | -6.51354200 | -0.86265000 | -0.30220700 |
| H | -6.78291800 | -3.02435400 | -0.19756200 |
| H | -5.94827600 | 1.20995100 | -0.44846900 |
| C | 3.76398400 | -1.40280300 | -0.11682400 |
| C | 4.73618600 | -2.45356600 | -0.13381400 |
| C | 4.23485900 | -0.06064700 | -0.24752800 |
| C | 6.07264300 | -2.20644400 | -0.20715800 |

| | | | |
|---|---|---|---|
| H | 4.38554100 | -3.47925700 | -0.06518800 |
| C | 5.56360900 | 0.20200500 | -0.33759200 |
| H | 3.53339200 | 0.76280600 | -0.31256000 |
| C | 6.51369200 | -0.86243700 | -0.30220900 |
| H | 6.78312600 | -3.02413200 | -0.19752300 |
| H | 5.94837100 | 1.21014600 | -0.44852000 |
| C | 0.00002000 | 2.07145600 | 1.86436800 |
| H | 0.87926900 | 2.16095700 | 2.51222900 |
| H | -0.87911300 | 2.16084900 | 2.51240300 |
| C | -0.00015700 | 3.15170400 | 0.81736700 |
| C | -1.20000300 | 3.65648500 | 0.31300300 |
| C | 1.19951500 | 3.65655300 | 0.31265700 |
| C | -1.20253900 | 4.62563800 | -0.67928400 |
| H | -2.14401900 | 3.31463000 | 0.73550200 |
| C | 1.20170600 | 4.62570600 | -0.67963300 |
| H | 2.14367300 | 3.31475000 | 0.73488000 |
| C | -0.00050300 | 5.10702700 | -1.18197600 |
| H | -2.14203500 | 5.02380100 | -1.04881200 |
| H | 2.14107300 | 5.02392000 | -1.04943400 |
| H | -0.00063600 | 5.87407800 | -1.94918900 |
| O | -7.75533000 | -0.49492200 | -0.37726000 |
| O | 7.75547200 | -0.49467600 | -0.37724400 |
| C | -8.83324500 | -1.44988200 | -0.36230300 |
| H | -8.80927800 | -2.02724800 | 0.56495600 |
| H | -9.74325200 | -0.85758100 | -0.40983200 |
| H | -8.76467800 | -2.10294300 | -1.23544200 |
| C | 8.83341200 | -1.44960700 | -0.36224700 |
| H | 9.74340400 | -0.85728200 | -0.40976900 |

| | | | |
|---|---|---|---|
| H | 8.80944200 | -2.02695500 | 0.56502200 |
| H | 8.76488100 | -2.10268600 | -1.23537500 |
